# Meta-Analysis of miRNA Variants Associated with Susceptibility to Autoimmune Disease

**DOI:** 10.1155/2021/9978460

**Published:** 2021-10-08

**Authors:** Jun Zhang, Handan Tan, Qingfeng Cao, Guannan Su, Peizeng Yang

**Affiliations:** The First Affiliated Hospital of Chongqing Medical University, Chongqing Key Lab of Ophthalmology, Chongqing Eye Institute, Chongqing Branch of National Clinical Research Center for Ocular Diseases, Chongqing, China

## Abstract

**Purpose:**

Various studies have shown an association between miRNA polymorphisms and susceptibility to autoimmune disease (AD); however, the results are inconclusive. To evaluate whether miRNA polymorphisms account for a significant risk of AD, a total of 87 articles, including 39431 patients and 56708 controls, were identified to estimate their association with 12 AD subtypes.

**Methods:**

Several electronic databases were searched to analyze population-based studies on the relationship between miRNA variants and AD risk. Fixed effects or random effect models were used in the meta-analysis for the risk assessment.

**Results:**

In our meta-analysis, miR-146a rs2910164/rs57095329 conferred a marginally elevated risk for AD (allele model, OR = 1.08, 95% CI: 1.01-1.15, *P* = 0.019; allele model, OR = 1.09, 95 CI: 1.05-1.15, *P* < 0.001, respectively). Furthermore, miR-196a2 rs11614913 was also associated with AD risk (allele model, OR = 0.92, 95% CI: 0.88-0.97, *P* = 0.001) as well as miR-499 rs3746444 (allele model, OR = 1.16, 95% CI: 1.03-1.29, *P* = 0.011). In addition, associations were observed between miR-149 rs2292832/miR-27a rs895819 and AD susceptibility in the overall population (allele model, OR = 1.15, 95% CI: 1.06-1.24, *P* < 0.001; allele model, OR = 1.11, 95% CI:1.01-1.22, *P* = 0.043, respectively).

**Conclusions:**

Evidence from our systematic review suggests that miR-146a, miR-196a2, miR-499, miR-149, and miR-27a polymorphisms are associated with susceptibility to AD.

## 1. Introduction

Autoimmune diseases (AD) are a spectrum of disorders initiated by impaired self-tolerance of the immune system and may lead to tissue destruction, chronic inflammation, and morbidity [1]. More than 80 types of AD have been confirmed and affect approximately 5-10% of the total population [2]. Of note, women constitute approximately 78% of those affected individuals and bear a disproportionate burden of the high morbidity [3]. Like many other complex diseases, AD are believed to arise from multiple environmental and genetic factors, both of which may be shared across many AD [4]. Common nonsteroidal anti-inflammatory drugs, immunosuppressants, and antitumor necrosis factor-alpha agent could be utilized for the treatment of various AD. While some patients did not respond to these treatments, suggesting other factors such as genetic background may account for this heterogeneity [5]. Association and linkage analysis in different populations have demonstrated that hereditary variation of AD has intrinsic commonality. Most of the mutations are typically located in either coding gene regions with an influence on protein function or noncoding gene regions, potentially affecting a targeted gene transcript. Additionally, recent studies suggested that functional variants occurring in microRNA sequences were associated with susceptibility to AD [6–15]. These findings indicate that variants in common miRNAs could be genetic markers of AD such as multiple sclerosis, rheumatoid arthritis, and ankylosing spondylitis, which highlighted a new paradigm for genetic susceptibility.

MicroRNAs (miRNAs) are endogenously generated single-stranded noncoding RNA molecules of about 22 nucleotides that play a pivotal role in regulating transcription and posttranscription of specific gene expression, including genes of the mammalian immune system [16]. Genetic ablation of the miRNA machinery, as well as various single nucleotide polymorphisms (SNPs) resulting in the loss or dysregulation of miRNAs (miRNA-SNPs), may affect immune development, differentiation, and response, which could ultimately lead to loss of immune tolerance and autoimmunity [16, 17]. Furthermore, functional miRNA-SNPs may act as potential biomarkers to predict clinical outcome or susceptibility of AD [18, 19]. Growing evidence indicated that studies on miRNA function have been moved to molecular mechanism level. miRNA could inhibit translation at the initiation step, likely involving the m7G cap structure or implicating the cap-binding protein eukaryotic initiation factor [20, 21]. Additionally, miRNA also inhibits actively translating polyribosomes or ribosome drop during ongoing translation [22, 23].

A robust quantification of the correlation regarding the miRNA-SNPs in patients with AD risk may increase our understanding whether genetic mutations in miRNA sequence are associated with immune-related diseases. Whether shared genetic variations may have similar effects on the risk of different AD or whether these effects are specific for certain AD has not yet been investigated at the genotype level. Although several meta-analyses have already addressed the impact of miRNA polymorphisms on AD risk [24–27], results have been controversial and often lacked sufficient statistical power. Apparently, a review of more recent studies is required to enhance the existing knowledge and clarify the observed inconsistencies. Using novel meta-analysis techniques, we readdressed this subject to evaluate the association between common miRNA-SNPs with susceptibility to AD.

## 2. Materials and Methods

### 2.1. Search Strategy

Studies reporting miRNA disease associations were retrieved from various databases including PubMed, Embase, Web of Science, Google Scholar, and the Chinese National Knowledge Infrastructure (CNKI, http://www.cnki.net/) registry, using the following keywords: “polymorphism,” “SNP,” “variant,” “genotype,” “autoimmune,” “immune-related,” “miRNA,” “microRNA,” and “microRNAs.” Each database was screened from the inception date to December 10, 2020. There were no restrictions as to language, ethnicity, or publication year. Additionally, the citations of retrieved articles were also manually scrutinized for original data sources. The Preferred Reporting Items for Systematic Reviews and Meta-Analyses (PRISMA) checklist including the page number for each item can be available in Supplementary Materials (available here).

### 2.2. Selection Criteria

Full length articles were reviewed for relevant keywords in the title, abstract, or keyword list. Publications were checked in the first round using the following inclusion criteria: (1) assessment of all miRNA genetic polymorphism association studies, (2) independent case-control study, and (3) having enough data to enable calculating odds ratios (OR) with 95% confidence intervals (95% CI). Articles in the category of reviews, meta-analysis, organizational guidelines, editorial letters, expert opinions, conference abstracts, case reports, and those with insufficient raw data (after contacting the corresponding author) were excluded to avoid duplication and erroneous weighting towards more frequently cited publications. Full text screening of all studies conforming to the above criteria was performed independently by two reviewers (J.Z. and H.T.). Any discrepancy was addressed with a third reviewer (Q.C.) to reach a consensus.

### 2.3. Data Extraction and Quality Assessment

A data extraction sheet based on a predetermined standard, including the first author, publication year, type of disease, country, ethnicity, genotyping methods, characteristics of cases and controls, Hardy-Weinberg equilibrium (HWE) in controls, and the modified Newcastle-Ottawa Quality Assessment Scale (NOS), was compiled for each selected study. A study with a NOS score of seven or more points was considered high quality, and those with nine points were ranked the most senior. A kappa value was calculated to compare the data retrieved by the two reviewers (J.Z. and H.T.) [28]. The Grading of Recommendations, Assessment, Development, and Evaluation (GRADE) system was adopted to evaluate the quality of evidence of the included studies [29]. After going through these quality checks, a final list of studies was produced.

### 2.4. Data Synthesis and Meta-Analysis

In our study, the term meta-analysis included our analysis of all miRNA SNPs in the combined AD groups. In the subgroup analysis of specific diseases, sometimes only one study could be found and as such would not meet the definition of a meta-analysis. Mantel-Haenszel OR with 95% CI was computed from the initial raw data; heterogeneity was measured by exploring the study-specific Cochran's *Q* value (*P* < 0.1, treated as significant level across all reviews) and quantitative Higgins's *I*^2^ statistic. When the *I*^2^ statistic was higher than 75%, 50%, and 25%, it represented large, moderate, and small heterogeneity, respectively. Thus, either the fixed effects model (*I*^2^ < 50% and *P* > 0.1) or random effects model (*I*^2^ ≥ 50% and *P* < 0.1) was utilized to measure the pooled ORs and 95% CIs. Furthermore, *I*^2^ offers advantages over Cochran's *Q* statistic and *I*^2^ is preferable to a test for heterogeneity in assessing inconsistency across studies [30]. A chi-square test was conducted in controls to evaluate the deviation from HWE. Subgroup analysis was used to test the influence of the categorical moderators. Additionally, metaregression was used to evaluate the contribution of different covariables to heterogeneity. Dummy variables were applied where features had three or more outcomes (for example, ethnicity, disease type, and genotype method). A sensitivity analysis was implemented to assess the influence of each study on the pooled effect size by taking out one study in each turn. Publication bias was tested by Begg's funnel plot and Egger's regression method. A *P* value less than 0.05 was treated as significant in these comparisons, and all statistical analyses were achieved by STATA V.12.0 software (Stata Corp LP, College Station, Texas, USA).

## 3. Results

### 3.1. General Characteristics of Selected Studies and Quality Assessment

**Figure 1:**
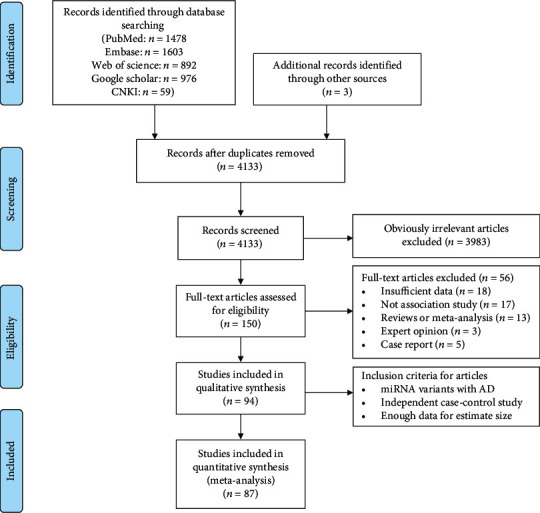
Flow diagram presenting the result of literature searching process in meta-analysis.

The initial search retrieved 1478 publications from PubMed, 1603 from Embase, 892 from the Web of Science, 976 from Google Scholar, and 59 from the CNKI. Three extra articles were obtained by scanning the references of preliminary papers [31–33]. The detailed step by step of our searching strategy is drawn as a PRISMA flowchart in Figure 1. 5011 articles were screened from the databases, and 878 duplicates were excluded. After a careful choice of papers following review of titles, abstracts, and key terms related to miRNA-SNP or AD, 3983 studies were deleted for not addressing neither miRNA-SNP nor AD, and 150 full-text articles were identified to be potentially relevant. Among them, 56 studies were eliminated since they did not contain sufficient genotype results (*n* = 18) or for not investigating an association between miRNA-SNP and AD risk (*n* = 17) or for being a review or meta-analysis (*n* = 13), expert opinion (*n* = 3), or case report (*n* = 5). Ultimately, 94 articles met all the inclusion criteria for our meta-analysis, of which 87 were eligible for quantitative analysis of risk estimates [6–15, 31–107]. Assessment of interinvestigator agreement using kappa values for the selected articles yielded values of 0.84 for PubMed, 0.88 for Embase, 0.90 for Web of Science, 0.91 for Google Scholar, and 1.0 for CNKI, suggesting a high level of agreement between our two reviewers.

**Figure 2:**
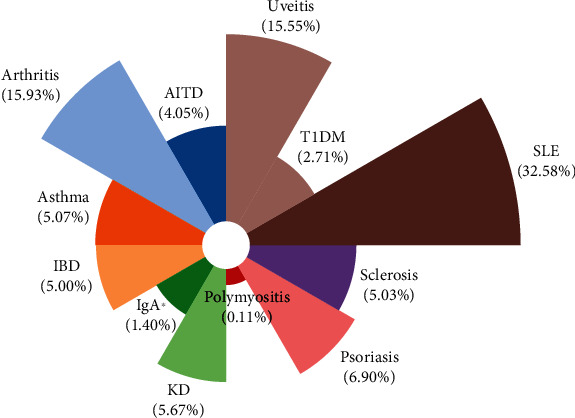
The distribution of total patients in the overall subgroups.

In our meta-analysis, AD were classified into twelve disease subgroups, including autoimmune thyroiditis (AITD), arthritis, asthma, systemic lupus erythematosus (SLE), uveitis, inflammatory bowel disease (IBD), Immunoglobulin A (IgA) nephropathy, Kawasaki disease (KD), sclerosis, type 1 diabetes mellitus (T1DM), polymyositis, and psoriasis based on the common syndrome and disease homogeneity risk [108]. We included asthma as an AD, because it is triggered not only by allergen exposure but also by other mechanisms, possibly autoreactive/autoimmune. This classification is further supported by the response to immunosuppressive drugs. The distribution of total patients in the overall subgroups is delineated in Figure 2, and the top five include SLE (32.6%), arthritis (15.9%), uveitis (15.5%), psoriasis (6.9%), and KD (5.7%). Furthermore, ethnic origins were categorized as Caucasian, East Asian, Hispanic, Middle East, and Oceanian. The basic characteristic of each study is shown in Table 1. Only SNPs with a minor allele frequency greater than 5% of the control population were included. The genotype frequencies of the controls in all studies, except seven articles, conformed to HWE (*P* > 0.05). Apart from two papers, the quality of the evidence generally received a score ranging from five to nine by the NOS criteria. All included studies were graded as “low” quality according to the GRADE profiler, except for one study [81] which was graded as “very low.” Low gradings were due to the observational design of studies, putting them at risk of bias, imprecision, and inconsistency.

### 3.2. Quantitative Data Synthesis and Meta-Analysis

87 articles describing 109 studies with 39431 patients and 56708 controls were finally included. Pooling these data, we estimated the miRNA-SNP risk for 23 AD and accomplished a meta-analysis of 12 AD subtypes into the case group. The following paragraphs discuss the epidemiological studies and summarize the genetic susceptibility to AD (Table 2, supplementary materials (available here)).

#### 3.2.1. miR-146a

**Figure 3:**
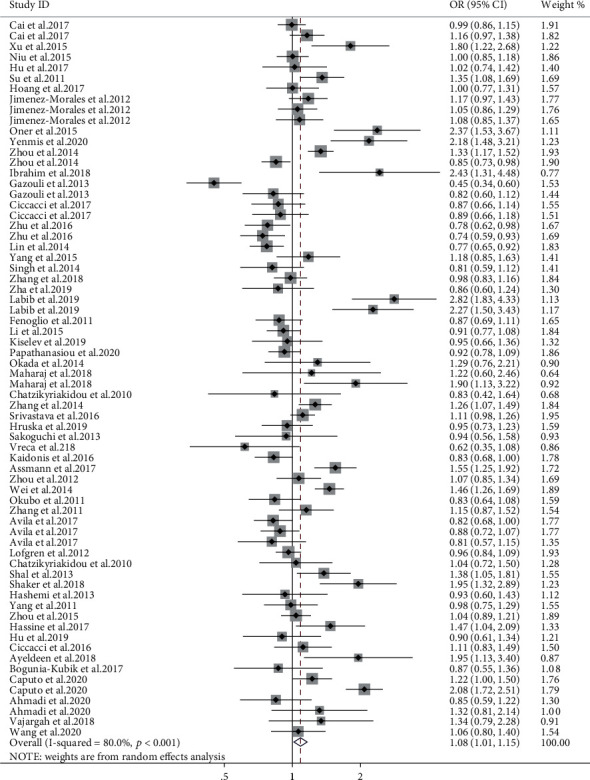
Assessment of the association between miR-146a rs2910164 polymorphism (G vs. C) with AD.

A total of four SNPs (rs2910164, rs57095329, rs2431697, and rs6864584) in the miR-146a gene were investigated from data retrieved from 71 studies. Meta-analysis indicated that the G allele of rs2910164 was positively associated with AD susceptibility in the overall population (allele model, OR = 1.08, 95% CI: 1.01-1.15, *P* = 0.019, Figure 3; dominant model, OR = 1.09, 95% CI: 1.01-1.20, *P* = 0.049). After stratifying by disease subtype, it was associated with a decreased risk of IBD (allele model, OR = 0.79, 95% CI: 0.65-0.97, *P* = 0.027; dominant model, OR = 0.78, 95% CI: 0.65-0.92, *P* = 0.001; recessive model, OR = 0.67, 95% CI: 0.50-0.88, *P* = 0.005). On the contrary, it was correlated with increased risk of arthritis (allele model, OR = 1.15, 95% CI: 1.01-1.31, *P* = 0.034; recessive model, OR = 1.17, 95% CI: 1.01-1.36, *P* = 0.048), with asthma (allele model, OR = 1.16, 95% CI: 1.03-1.31, *P* = 0.014; dominant model, OR = 1.25, 95% CI: 1.02-1.54, *P* = 0.029), with uveitis (allele model, OR = 1.44, 95% CI: 1.14-1.81, *P* = 0.002; dominant model, OR = 1.26, 95% CI: 1.13-1.40, *P* < 0.001; recessive model, OR = 1.73, 95% CI: 1.21-2.47, *P* = 0.003), and with psoriasis (allele model, OR = 1.15, 95% CI: 1.05-1.25, *P* = 0.001; dominant model, OR = 1.38, 95% CI: 1.13-1.69, *P* = 0.002). A stratified analysis by ethnicity revealed a significant increase in the risk of AD in the Middle East population (especially in the allele model, OR = 1.66, 95% CI: 1.35-2.04, *P* < 0.001; dominant model, OR = 2.54, 95% CI: 1.60-4.02, *P* < 0.001; recessive model, OR = 2.13, 95% CI: 1.54-2.95, *P* < 0.001), but a decreased risk in the Caucasian and Oceanian groups using the recessive model (OR = 0.86, 95% CI: 0.75-0.99, *P* = 0.036; OR = 0.78, 95% CI: 0.61-0.99, *P* = 0.037, respectively). Subgroup meta-analysis by methodological quality of the studies as ranked by the NOS scale revealed no significant positive association in neither the high-quality studies nor the low-quality studies (shown in supplementary materials (available here)).

Furthermore, an elevated risk of AD was found in subjects with the rs57095329 G allele model (OR = 1.09, 95 CI: 1.05-1.15, *P* < 0.001). In addition, a stratified analysis based on disease subtype showed that this variant conferred an increased risk of SLE in the allele model and a decreased risk of sclerosis in the recessive model. In the stratified analysis by ethnicity, a significant relationship was detected in the East Asian and Middle East groups (shown in supplementary materials (available here)).

Moreover, pooled results showed that the C allele of rs2431697 was associated with a significantly decreased risk of AD in the overall population (allele model, OR = 0.77, 95% CI: 0.71-0.84, *P* < 0.001; dominant model, OR = 0.74, 95% CI: 0.56-0.98, *P* = 0.037; recessive model, OR = 0.76, 95% CI: 0.62-0.92, *P* = 0.006). Based on the disease subtype, an obvious association was found in SLE among three genetic models. Our stratified analysis results revealed a significant association in the East Asian and Caucasian groups (shown in supplementary materials (available here)).

Lastly, meta-analysis suggested that the rs6864584 C allele was associated with a decreased risk of AD in the total population (allele model, OR = 0.83, 95% CI: 0.69-0.99, *P* = 0.038; dominant model, OR = 0.82, 95% CI: 0.68-0.99, *P* = 0.039). Similar results were found in uveitis patients (shown in supplementary materials (available here)).

#### 3.2.2. miR-196a2

**Figure 4:**
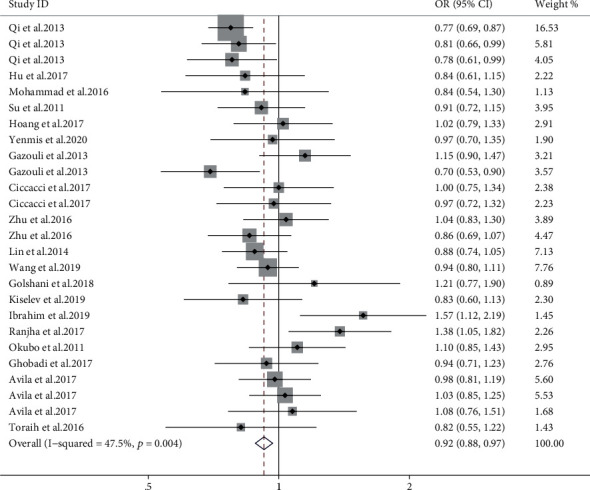
Estimation of the association between miR-196a2 rs11614913 polymorphism (T vs. C) with AD.

Combined results of 26 studies revealed that the T allele of miR-196a2 rs11614913 was associated with a lower risk of AD (allele model, OR = 0.92, 95% CI: 0.88-0.97, *P* = 0.001, Figure 4; dominant model, OR = 0.92, 95% CI: 0.86-0.98, *P* = 0.017; recessive model, OR = 0.87, 95% CI: 0.81-0.95, *P* = 0.002). Furthermore, there was a reduced risk of uveitis in the three genetic models (allele model, OR = 0.80, 95% CI: 0.73-0.87, *P* < 0.001; dominant model, OR = 0.74, 95% CI: 0.64-0.84, *P* < 0.001; recessive model, OR = 0.74, 95% CI: 0.62-0.87, *P* < 0.001), but an increased risk of T1DM in the allele and recessive model. The stratified analysis results demonstrated that rs11614913 T was significantly related with a decreased risk of AD in the East Asian population (shown in supplementary materials (available here)). Subgroup meta-analysis by the NOS scale revealed a significant negative association in the high-quality studies (OR = 0.90, 95% CI: 0.86-0.95, *P* < 0.001) but not the low-quality studies (shown in supplementary materials (available here)).

#### 3.2.3. miR-499

**Figure 5:**
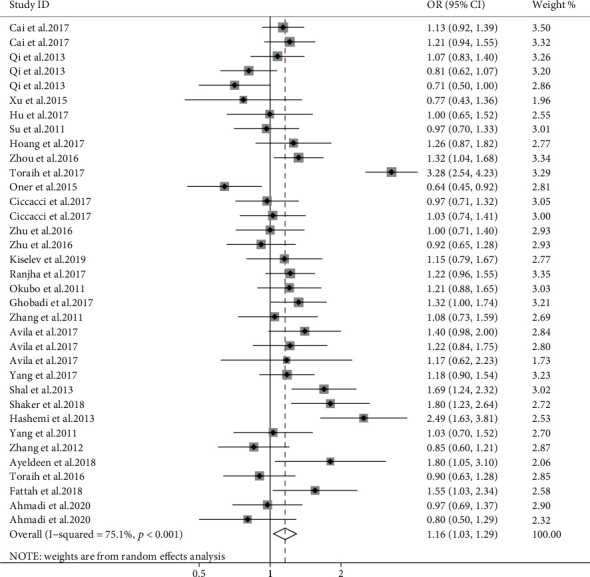
Evaluation of the association between miR-499 rs3746444 polymorphism (C vs. T) with AD.

Meta-analysis of 35 case-control studies showed miR-499 rs3746444 is a predisposing cause of AD (allele model, OR = 1.16, 95% CI: 1.03-1.29, *P* = 0.011, Figure 5; dominant model, OR = 1.16, 95% CI: 1.09-1.24, *P* < 0.001; recessive model, OR = 1.47, 95% CI: 1.30-1.66, *P* < 0.001). In the subgroup analysis by disease subtypes, rs3746444 polymorphisms increased susceptibility for arthritis and asthma (allele model, OR = 1.29, 95% CI: 1.15-1.44, *P* < 0.001; dominant model, OR = 1.24, 95% CI: 1.09-1.42, *P* = 0.001; recessive model, OR = 1.54, 95% CI: 1.23-1.92, *P* < 0.001; allele model, OR = 1.56, 95% CI: 1.36-1.77, *P* < 0.001; dominant model, OR = 1.48, 95% CI: 1.26-1.74, *P* < 0.001; recessive model, OR = 2.80, 95% CI:2.03-3.88, *P* < 0.001, respectively). On the other hand, a reduced susceptibility was observed for uveitis (allele model, OR = 0.83, 95% CI: 0.72-0.97, *P* = 0.017; recessive model, OR = 0.59, 95% CI: 0.39-0.89, *P* = 0.012). A stratified analysis hinted that rs3746444 C delivered an increased risk of AD in the Hispanic and Middle East region (shown in supplementary materials (available here)). Additionally, subgroup meta-analysis based on the NOS scale revealed a significant positive association in the high-quality studies (OR = 1.21, 95% CI: 1.03–1.41, *P* = 0.018) but not the low-quality studies (shown in supplementary materials (available here)).

#### 3.2.4. Other miRNAs

In addition, a significantly increased *risk* was observed between miR-149 rs2292832/miR-27a rs895819/miR-182 rs76481776/miR-23a rs3745453 and AD susceptibility in the overall population (allele model, OR = 1.15, 95% CI: 1.06-1.24, *P* = 0.001; dominant model, OR = 1.13, 95% CI: 1.01-1.26, *P* = 0.027; allele model, OR = 1.11, 95% CI: 1.01-1.22, *P* = 0.043; recessive model, OR = 1.28, 95% CI:1.05-1.55, *P* = 0.013; allele model, OR = 1.62, 95% CI: 1.43-1.82, *P* < 0.001; dominant model, OR = 1.63, 95% CI: 1.43-1.86, *P* < 0.001; recessive model, OR = 2.22, 95% CI: 1.48-3.34, *P* < 0.001; allele model, OR = 1.68, 95% CI: 1.39-2.05, *P* < 0.001; dominant model, OR = 1.61, 95% CI: 1.25-2.08, *P* < 0.001; recessive model, OR = 2.82, 95% CI: 1.84-4.32, *P* < 0.001, respectively). Subgroup analysis showed an increased risk of arthritis and asthma but a reduced risk of IBD with rs2292832 polymorphisms. Moreover, there was no apparent correlation between other miRNAs and AD susceptibility (shown in supplementary materials (available here)).

### 3.3. Heterogeneity Test and Meta-regression Analysis

The merged results revealed conspicuous heterogeneities in the combined disease groups (heterogeneity: *I*^2^ = 47.5%-76.3%, *P* < 0.001, shown in Table 2 and supplementary materials (available here)). To further explore the source of heterogeneity, we performed a univariate metaregression analysis based on the random effects model. Several covariate factors such as disease subtype, genotypic method, ethnicity, mean age, the number of cases, and percentage of females in cases were evaluated using three genetic models (allelic, dominant, and recessive, Table 3). In our combined analysis, a statistically significant effect on the summary ORs through disease subtype and ethnicity was discovered (allele model, *P* = 0.031, OR = 1.02-1.48; *P* = 0.005, OR = 1.25-3.42), which could partly account for the variation of heterogeneity (adjusted *R*^2^ = 35.16%, adjusted *R*^2^ = 45.71%, respectively) (shown in Table 3).

### 3.4. Sensitivity Analysis

**Figure 6:**
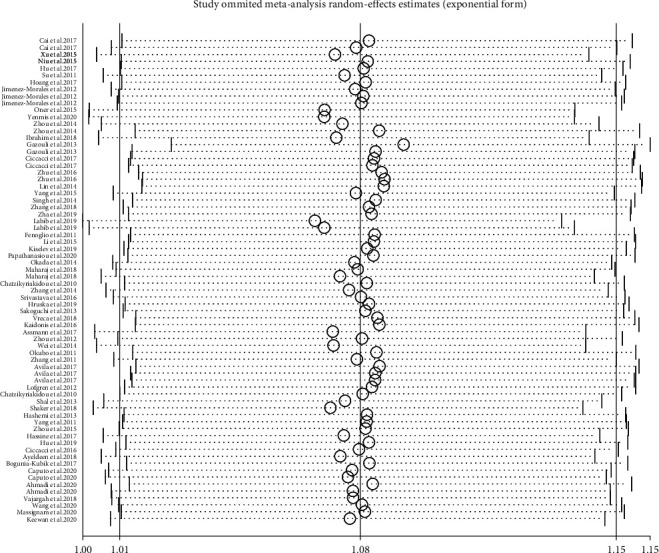
Assessment of the sensitivity analysis between miR-146a rs2910164 polymorphism (G vs. C) with AD.

To evaluate the effect of publication on the robustness of our pooled effect estimate, a sensitivity analysis was performed by deleting each study once at a time in the three genetic models. As a result, the summary OR did not make any difference on the overall risk estimates, which suggested that our meta-analysis was reliable (Figure 6, rs2910164 G vs. C).

### 3.5. Publication Bias

**Figure 7:**
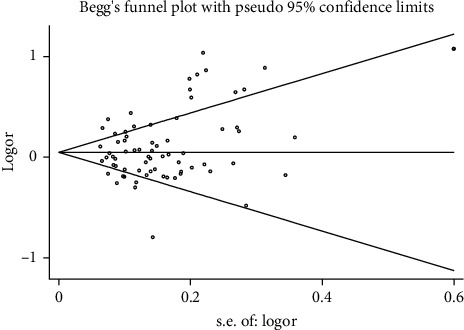
Estimation of the publication bias between miR-146a rs2910164 polymorphism (G vs. C) with AD.

Publication bias was investigated according to the test of Begg's funnel plot and Egger's regression analysis. Due to the relatively small number of included studies, publication bias analysis could not be carried out for miR-155 rs767649, miR-125a rs12976445, miR-182 rs76481776, miR-585 rs62376935, miR-23a rs3745453, miR-106a rs3747440, miR-122 rs17669, miR-124a rs531564, and miR-137 rs1625579. As for the other miRNA polymorphisms, no evidence of publication bias was observed neither with the Begg's funnel plot nor the Egger's test (Figure 7, rs2910164 G vs. C), which implies that our results were statistically robust (*P* > 0.05, Table 4).

## 4. Discussion

In this systematic meta-analysis of 87 case-control studies, 17 SNPs from 14 miRNA genes were shown to be associated with the susceptibility to AD. Five miRNAs with eight variants were shared across AD and may play potential roles in the pathogenesis of AD (shown in supplementary materials (available here)).

A mounting body of evidence has demonstrated that miRNAs cause gene silencing by degrading targeted mRNAs or by inhibiting translation. Common variants in miRNAs can rearrange a broad range of biological processes by influencing the processing or altering target selection of miRNAs [109], thus dysregulating miRNA expression, which may be involved in the development of a wide range of diseases including AD. Although several meta-analysis studies have shown the association between miRNA variants with AD, most of them only discussed a single SNP.

Previous studies have demonstrated that miRNAs may play an important role in the regulation of the immune system. The well-known miR-146a, which is a negative regulator of the NF-*κ*B activation pathway, can modulate mRNAs that encode proteins involved in the control of innate or adaptive immune responses [110]. SNP rs2910164, which is located in the stem sequence of the miR-146a precursor, can directly influence the expression of miR-146a [111]. Five studies presented independent evidence that the rs2910164 G allele was not correlated with arthritis [24–26, 112, 113]. Furthermore, a previous meta-analysis [108] also showed no association with inflammatory arthritis, IBD, and a uveitis subgroup. Our meta-analysis, however, did suggest that the G allele was protective against IBD and that it was a risk factor for arthritis, asthma, uveitis, and psoriasis. The remarkable difference between our data and the earlier meta-analyses may be due to the fact that we included more studies and therefore had a higher number of patients. rs57095329, which is located in the promoter region of the miR-146a gene, has been shown to induce the expression of miR-146a by altering its binding affinity with Ets-1 [62]. Furthermore, individuals containing the risk G allele tended to express a lower level of miR-146a in Asian patients, and further functional studies showed that it was a negative regulator of the IFN pathway. Our meta-analysis confirmed earlier studies [112, 113], showing that rs57095329 G was a high-risk factor for SLE but not for other AD in East Asian regions. These pooled results could be explained by the disease-specific influence on SLE. Moreover, recent SLE GWASs have identified the disease-related SNP-rs2431697, which lies upstream of the miR-146a gene, and showed that the C allele conferred protective susceptibility to SLE in Asians and Caucasians [114, 115]. Compared with a meta-analysis performed by earlier by others [112], our meta-analysis included more studies and a larger sample size and indicated that the C allele was protective against SLE in Asians and Caucasians, whereas the latter study was confined to an Asian population.

miR-146a rs6864584, which is positioned in the miR-146a precursor promoter region, also affects the expression of miR-146a [62]. Recent studies could not detect an association between rs6864584 C/T and KD or asthma [44, 63]. Our meta-analysis is the first to confirm the association between rs6864584 and uveitis.

The transcript variant (rs11614913) of miR-196a2 precursor has been reported to influence the efficient processing of mature miR-196a2 and the expression of its target gene [116]. Our analysis showed that the T allele conferred protection against uveitis. A decreased risk of miR-196a2 variants has been reported with T1DM in children and adolescents, but rs11614913 genotypes were not shown to affect miRNA expression [79]. An allele mutation of rs11614913 from C to T was detected in a majority of glioma tissues, but no association was discovered with the genotype [117]. In other words, the differential expression of miR-196a2 was probably not mediated by rs11614913 itself but by other factors.

Studies have confirmed that miR-499 rs3746444 is located in the pre-miRNA region and influences the binding of target mRNAs to 3p mature miRNA [118]. mir-499 targets the IL-17 receptor B, IL-6, and other cytokines, all of which play an important role in the pathogenesis of RA [119]. An miR-499 mutation, rs3746444, was shown to be associated with RA as well as with disease severity in Egyptian patients [43]. The CC genotype and C allele of this variant also confer genetic predisposition to RA in Iranians. The association between miR-499 polymorphisms and RA could not be confirmed in Chinese patients [72]. Our combined results not only showed that the rs3746444 C allele is associated with an increased risk for arthritis but also showed that it was protective for uveitis in Middle East populations. In agreement with a previous meta-analysis [27], we also showed an association between the CC genotype and asthma.

The rs2292832 T>C mutation of miR-149 may affect its expression and susceptibility to disease [40, 63]. miR-149 is a proapoptotic miRNA that affects the expression of the Akt1 and E2F1 gene, which has been shown to promote cell growth and cell cycle progression in IBD-associated colorectal cancer [120]. Our meta-analysis revealed significant associations between rs2292832 and the risk of developing arthritis, asthma, and IBD. The available data was focused on Asian populations, and it would be interesting to investigate a possible association between miR-149 variants and AD in other ethnic groups.

We did not find associations for several miRNA variants including miR-27a, miR-155, miR-125a, miR-182, miR-585, miR-23a, miR-106a, miR-122, miR-124a, and miR-137. Our analysis did reveal an association between miR-182 rs76481776 and uveitis susceptibility as well as an association between miR-23a rs3745453 and sclerosis.

Understanding the specific miRNA-regulated genetic networks and molecular mechanisms by which miRNAs participate in the immune system is a promising area of research and promotes their clinical application. The diagnostic and therapeutic manners of miRNAs have long been acknowledged, which are regarded as clinical biomarkers for monitoring disease evolution during treatment [121]. In the future, miRNAs in biofluids such as saliva could be excellent biomarkers, because their collection is noninvasive and easy to be performed [122]. Thus, it is urgent to explore the molecular role of miRNAs in the pathophysiology of AD and to evaluate possible clinical and future implications for a personalized approach.

Although we retrieved all current available studies, some limitations of our analysis should be mentioned. First, the disproportionate numbers of cases (range: 0.11%-32.58%) in different AD subtypes might have yielded different sample sizes; thus, the statistical power may show potential heterogeneity. Second, several studies were mainly focused on asthma and Kawasaki disease risk relationship with younger children (younger than five years old), and this may inevitably produce age bias. Third, several groups only contained two studies, which makes it difficult to generalize results, suggesting that larger sample sizes are needed to validate the relationship. Fourth, the current research should be registered in the PROSPERO or Cochrane system, and we hope to do so in future but for the time being would like to mention that our meta-analysis was performed strictly in accordance with the process of systematic review. In addition, the association level identified by current studies was low because of imprecision according to GRADE profiler. Finally, more attention should be made concerning a possible gender bias. However, metaregression did not show that the gender ratio affected our pooled results.

## 5. Conclusions

Taken together, our meta-analysis provides evidence that miR-146a, miR-196a2, miR-499, miR-149, and miR-27a polymorphisms are associated with AD susceptibility. Some polymorphisms are shared by several AD in certain ethnic groups and/or geographic locations. Some miRNA polymorphisms show protection in some diseases and an increased susceptibility in others. These results provide further support to the complexity of autoimmune disease and suggest that prevention and treatment should be tailored for each specific immune disorder.

## Figures and Tables

**Table 1:** The general characteristics of all studies included in our meta-analysis.

Refs	Year	Disease	Group	Mean age	Female (%)	Country	Ethnicity	Case/control	Typing method	NOS score	MAF control	HWE control
Srivastava et al. [78]	2017	GD	AITD	36.92	69.5	China	East Asian	678/918	LDR	6	0.42	Yes
Srivastava et al. [78]	2017	HD	AITD	35.03	87.8	China	East Asian	344/918	LDR	6	0.42	Yes
Wang and Pan [25]	2015	AS	Arthritis	34.88	25.9	China	East Asian	102/105	Sequence	7	0.35	Yes
Zhang et al. [8]	2015	AS	Arthritis	—	21.3	China^∗^	East Asian	609/616	Sequence	6	0.41	Yes
Gazouli et al. [54]	2017	Asthma	Asthma	6.20	52.3	China	East Asian	124/206	TaqMan	7	0.36	Yes
Higgins et al. [30]	2011	Asthma	Asthma	35.4	52.9	China	East Asian	219/513	RFLP	8	0.45	Yes
Zha et al. [44]	2017	Asthma	Asthma	41.8	58.5	Korea	East Asian	341/170	SNaPShot	6	0.38	Yes
Fenoglio et al. [6]	2012	Asthma	Asthma	8.40	41.0	Mexico	Hispanic	402/531	TaqMan	7	0.34	Yes
Fenoglio et al. [6]	2012	SLE	SLE	11.61	83.0	Mexico	Hispanic	367/531	TaqMan	7	0.34	Yes
Fenoglio et al. [6]	2012	JRA/JIA	Arthritis	8.70	59.0	Mexico	Hispanic	210/531	TaqMan	7	0.34	Yes
El-Shal et al. [43]	2015	BD	Uveitis	34.8	50.0	Turkey	Middle East	100/145	RFLP	6	0.33	NO
Senousy et al. [12]	2020	BD	Uveitis	33.4	51.3	Turkey	Middle East	117/220	RFLP	7	0.32	Yes
Ghobadi et al. [68]	2014	BD	Uveitis	32.1	16.2	China	East Asian	809/1132	RFLP	9	0.36	Yes
Ghobadi et al. [68]	2014	VKH	Uveitis	33.2	46.8	China	East Asian	613/1132	RFLP	9	0.36	Yes
Ahmed et al. [94]	2018	BD	Uveitis	34.4	14.9	Egypt	Middle East	47/50	TaqMan	6	0.44	Yes
Abdelaleem et al. [46]	2013	CD	IBD	51.3	55.4	Greece	Caucasian	242/300	RFLP	7	0.18	Yes
Abdelaleem et al. [46]	2013	UC	IBD	50.9	51.9	Greece	Caucasian	210/300	RFLP	7	0.18	Yes
Zhou et al. [77]	2017	CD	IBD	30.0	51.3	Italy	Caucasian	244/298	TaqMan	6	0.27	Yes
Zhou et al. [77]	2017	UC	IBD	37.4	41.1	Italy	Caucasian	206/298	TaqMan	6	0.27	Yes
Dong et al. [27]	2016	CD	IBD	43.8	48.0	China	East Asian	227/450	RFLP	7	0.44	Yes
Dong et al. [27]	2016	UC	IBD	41.7	46.9	China	East Asian	241/450	RFLP	7	0.44	Yes
Zhu et al. [36]	2014	IgA^∗^	IgA^∗^	11.8	41.8	China	East Asian	404/711	LDR	8	0.44	Yes
Aleman-Avila et al. [61]	2015	IgA^∗^	IgA^∗^	33.5	52.0	China	East Asian	145/179	Roche	7	0.34	Yes
Che et al. [33]	2014	JRA/JIA	Arthritis	11.0	11.3	India	East Asian	150/216	RFLP	6	0.27	Yes
Fattah et al. [93]	2018	KD	KD	28.4	31.4	China	East Asian	532/616	TaqMan	9	0.39	Yes
Yang et al. [35]	2019	KD	KD	—	33.3	China	East Asian	120/126	RFLP	6	0.40	Yes
Atkins et al. [29]	2019	MS	Sclerosis	31.3	84.2	Egypt	Middle East	76/120	TaqMan	6	0.45	Yes
Atkins et al. [29]	2019	SLE	SLE	32.3	90.0	Egypt	Middle East	80/120	TaqMan	6	0.45	Yes
Hu and Daly [4]	2011	MS	Sclerosis	46.3	69.4	Italy	Caucasian	346/339	TaqMan	7	0.26	NO
Inoue et al. [57]	2015	MS	Sclerosis	32.6	72.8	China	East Asian	525/568	SNaPShot	7	0.44	Yes
Yu et al. [92]	2019	MS	Sclerosis	27.4	66.1	Russia	Caucasian	109/424	TaqMan	8	0.22	Yes
Wang et al. [10]	2020	Osteoarthritis	Arthritis	66.6	79.4	Greece	Caucasian	950/738	RFLP	6	0.22	Yes
Zhou et al. [73]	2014	Polymyositis	Polymyositis	58.7	60.9	Japan	East Asian	44/107	RFLP	3	0.28	NO
Vreca et al. [71]	2018	PsA	Arthritis	50.3	46.0	Caucasian	Caucasian	38/32	RFLP	6	0.36	Yes
Vreca et al. [71]	2018	PsA	Arthritis	50.3	46.0	Indian	East Asian	62/84	RFLP	6	0.36	Yes
Ciccacci et al. [86]	2020	Psoriasis	Psoriasis	—	—	Italy	Caucasian	393/600	TaqMan	7	0.30	Yes
Ciccacci et al. [86]	2020	PsA	Arthritis	—	—	Italy	Caucasian	424/600	TaqMan	7	0.30	Yes
Desai and Brinton [3]	2010	PsA	Arthritis	48.5	34.5	Greece	Caucasian	29/66	RFLP	6	0.27	NO
Jimeneze-Morales et al. [7]	2014	Psoriasis	Psoriasis	32.1	45.3	China	East Asian	521/582	RFLP	7	0.42	Yes
Golshani et al. [69]	2016	Psoriasis	Psoriasis	—	49.0	Sweden	Caucasian	1546/1526	TaqMan	6	0.21	Yes
Nottrott et al. [22]	2019	Psoriasis	Psoriasis	49.7	—	Czech	Caucasian	241/516	RFLP	8	0.22	Yes
Zhang et al. [72]	2013	SS	Sclerosis	—	—	Japan	East Asian	107/52	RFLP	1	0.29	NO
Luo et al. [62]	2018	SS	Sclerosis	57.7	86.3	Serbia	Caucasian	102/66	Sequence	6	0.17	Yes
Ayeldeen et al. [90]	2016	T1DM	T1DM	43.6	48.5	Australia	Oceanian	419/823	MALDI-TOF	6	0.23	Yes
Wei et al. [76]	2017	T1DM	T1DM	34.3	49.7	Brazil	Hispanic	431/405	TaqMan	7	0.33	Yes
Leng et al. [65]	2012	Fuchs	Uveitis	36.4	46.6	China	East Asian	219/612	RFLP	9	0.36	Yes
Lofgren et al. [67]	2014	PU	Uveitis	9.60	53.5	China	East Asian	520/1204	RFLP	9	0.37	Yes
Lin et al. [45]	2011	UC	IBD	40.2	43.5	Japan	East Asian	170/403	RFLP	8	0.40	Yes
Takuse et al. [41]	2011	SLE	SLE	34.5	94.4	China	East Asian	213/209	RFLP	7	0.36	Yes
Trinh et al. [52]	2017	SLE	SLE	39.9	94.3	Mexico	Hispanic	407/486	TaqMan	8	0.32	Yes
Trinh et al. [52]	2017	RA	Arthritis	51.8	91.7	Mexico	Hispanic	410/486	TaqMan	8	0.32	Yes
Trinh et al. [52]	2017	GD	AITD	36.2	88.9	Mexico	Hispanic	81/486	TaqMan	8	0.32	Yes
Wang et al. [58]	2012	SLE	SLE	—	—	Sweden	Caucasian	1109/1428	TaqMan	7	0.24	Yes
Hassine et al. [75]	2010	RA	Arthritis	60.8	80.0	Greece	Caucasian	136/147	RFLP	6	0.28	Yes
Sheng et al. [34]	2013	RA	Arthritis	38.4	—	Egypt	Middle East	217/245	RFLP	7	0.30	NO
Su et al. [39]	2018	RA	Arthritis	39.5	84.6	Egypt	Middle East	104/112	TaqMan	9	0.32	Yes
Singh et al. [42]	2013	RA	Arthritis	38.4	46.9	Iran	Middle East	104/110	RFLP	7	0.25	Yes
Burmester and Pope [5]	2011	RA	Arthritis	48.0	41.7	China	East Asian	208/240	RFLP	6	0.37	Yes
Qi et al. [64]	2015	RA	Arthritis	54.5	76.7	China	East Asian	598/821	SNPscan	9	0.42	Yes
Li et al. [66]	2017	RA	Arthritis	—	91.0	Tunisian	Middle East	165/150	MS-PCR	8	0.32	Yes
Li et al. [40]	2019	RA	Arthritis	39.0	81.7	China	East Asian	126/102	Roche	6	0.33	Yes
Ibrahim et al. [79]	2016	RA	Arthritis	54.1	76.5	Italy	Caucasian	192/298	TaqMan	7	0.27	Yes
Sakoguchi et al. [81]	2018	RA	Arthritis	39.5	84.6	Egypt	Middle East	52/56	TaqMan	8	0.32	Yes
Cai et al. [87]	2017	RA	Arthritis	—	—	Poland	Caucasian	111/130	RFLP	6	0.18	Yes
Maharaj et al. [80]	2020	RA	Arthritis	48.2	73.0	Iran	Middle East	89/237	T-ARMS-PCR	8	0.32	Yes
Maharaj et al. [80]	2020	SLE	SLE	45.2	92.0	Iran	Middle East	50/237	T-ARMS-PCR	8	0.32	Yes
Chen et al. [24]	2015	SLE	SLE	34.0	93.9	China^∗^	East Asian	1047/1205	MALDI-TOF	7	0.18	Yes
Chen et al. [24]	2015	SLE	SLE	34.7	91.5	China^∗^	East Asian	2202/2208	MALDI-TOF	7	0.17	Yes
Chen et al. [24]	2015	SLE	SLE	33.1	93.3	China^∗^	East Asian	1307/6038	MALDI-TOF	7	0.17	Yes
Humphreys et al. [21]	2015	SLE	SLE	36.0	89.4	China	East Asian	322/353	RFLP	7	0.19	Yes
Assmann et al. [85]	2020	MS	Sclerosis	31.3	84.2	Egypt	Middle East	114/152	TaqMan	6	0.34	Yes
Okubo et al. [53]	2011	SLE	SLE	—	—	China^∗^	East Asian	2352/1080	Sequence	6	0.16	Yes
Okubo et al. [53]	2011	SLE	SLE	—	—	China^∗^	East Asian	1152/1152	TaqMan	6	0.20	Yes
Okubo et al. [53]	2011	SLE	SLE	—	—	Thailand	East Asian	464/982	TaqMan	6	0.23	Yes
Yang et al. [56]	2012	SLE	SLE	35.8	89.7	China^∗^	East Asian	858/967	MALDI-TOF	7	0.20	Yes
Ranjha et al. [47]	2018	BD	Uveitis	—	—	Iran	Middle East	100/100	T-ARMS-PCR	8	0.19	Yes
Shaker et al. [37]	2020	BD	Uveitis	33.6	13.1	Egypt	Middle East	130/131	TaqMan	7	0.32	Yes
Hu et al. [49]	2020	AS	Arthritis	28.4	29.0	China	East Asian	200/200	RFLP	6	0.47	Yes
Zare-Karizi et al. [55]	2013	BD	Uveitis	33.5	13.6	China	East Asian	859/1685	RFLP	9	0.45	Yes
Zare-Karizi et al. [55]	2013	VKH	Uveitis	35.5	44.5	China	East Asian	400/400	RFLP	9	0.48	Yes
Zare-Karizi et al. [55]	2013	AAU^+^as^+^	Uveitis	39.5	24.9	China	East Asian	209/400	RFLP	9	0.48	Yes
Zhou et al. [74]	2016	Asthma	Asthma	9.70	45.8	Egypt	Middle East	96/96	TaqMan	7	0.30	Yes
Niu et al. [9]	2019	KD	KD	28.4	31.4	China	East Asian	531/623	TaqMan	6	0.47	Yes
Yu et al. [60]	2018	MS	Sclerosis	30.0	75.0	Iran	Middle East	80/80	T-ARMS-PCR	7	0.34	Yes
Yang et al. [70]	2019	T1DM	T1DM	13.0	58.0	Egypt	Middle East	150/150	T-ARMS-PCR	7	0.31	Yes
Labib et al. [38]	2017	UC	IBD	35.5	40.6	India	East Asian	197/441	RFLP	6	0.22	Yes
Ridolfi et al. [59]	2017	UC	IBD	35.9	60.0	Iran	Middle East	210/212	RFLP	7	0.39	Yes
Okada et al. [82]	2016	RA	Arthritis	46.1	88.4	Egypt	Middle East	95/200	TaqMan	6	0.28	Yes
Ahmadi et al. [89]	2016	Asthma	Asthma	44.9	39.8	China	East Asian	591/621	MALDI-TOF	8	0.11	Yes
Toraih et al. [91]	2017	Asthma	Asthma	9.7	45.8	Egypt	Middle East	211/330	TaqMan	7	0.35	Yes
Li et al. [26]	2017	RA	Arthritis	51.9	86.0	China	East Asian	386/576	TaqMan	6	0.12	Yes
Hu et al. [63]	2013	RA	Arthritis	54.6	71.5	China	East Asian	206/466	MALDI-TOF	8	0.14	Yes
Chatzikyriakidou et al. [84]	2018	RA	Arthritis	41.7	86.0	Egypt	Middle East	100/100	RFLP	7	0.31	Yes
Tang et al. [31]	2019	KD	KD	28.4	31.4	China	East Asian	507/612	TaqMan	6	0.19	Yes
Ciccacci et al. [88]	2015	RA	Arthritis	49.0	—	China	East Asian	186/120	RFLP	8	0.28	Yes
Hruska et al. [32]	2017	GD	AITD	—	—	Japan	East Asian	118/76	RFLP	7	0.36	Yes
Hruska et al. [32]	2017	HD	AITD	—	—	Japan	East Asian	141/76	RFLP	7	0.36	Yes
Oner et al. [51]	2014	AAU + AS+	Uveitis	39.3	34.0	China	East Asian	230/650	RFLP	9	0.08	Yes
Oner et al. [51]	2014	AAU + AS-	Uveitis	39.3	34.0	China	East Asian	240/650	RFLP	9	0.08	Yes
Hussein et al. [83]	2014	BD	Uveitis	33.6	14.2	China	East Asian	400/600	RFLP	9	0.07	Yes
Hussein et al. [83]	2014	VKH	Uveitis	39.3	47.6	China	East Asian	900/1800	RFLP	9	0.07	Yes
Shaker et al. [48]	2014	GD	AITD	34.5	84.8	Japan	East Asian	155/118	RFLP	7	0.13	Yes
Shaker et al. [48]	2014	HD	AITD	37.1	82.0	Japan	East Asian	151/118	RFLP	7	0.13	Yes
Zhang et al. [50]	2013	MS	Sclerosis	40.3	73.3	Italy	Caucasian	399/420	TaqMan	8	0.24	Yes
Petersen et al. [23]	2018	KD	KD	28.4	31.4	China	East Asian	527/622	TaqMan	7	0.06	Yes
Papathanasiou et al. [11]	2020	MS	Sclerosis	31.2	77.8	Egypt	Middle East	108/104	TaqMan	6	0.35	Yes
Caputo et al. [95]	2020	SLE	SLE	31.8	93.8	Egypt	Middle East	65/40	TaqMan	8	0.43	Yes
Chatzikyriakidou et al. [96]	2020	T1DM	T1DM	42.1	50.3	Brazil	Hispanic	195/215	TaqMan	8	0.27	Yes
Bogunia-Kubik et al. [97]	2020	CD	IBD	36.0	56	America	Caucasian	19/23	TaqMan	6	0.22	Yes

GD: Graves' disease; AITD: autoimmune thyroid disease; HD: Hashimoto's disease; AS: ankylosing spondylitis; SLE: systemic lupus erythematosus; JRA/JIA: juvenile idiopathic/rheumatoid arthritis; BD: Behcet's disease; VKH: Vogt-Koyanagi-Harada; CD: Crohn's disease; UC: ulcerative colitis; IBD: inflammatory bowel disease; IgA^∗^: IgA nephropathy; KD: Kawasaki disease; MS: multiple sclerosis; PsA: psoriatic arthritis; SS: systemic sclerosis; T1DM: type 1 diabetes mellitus; PU: pediatric uveitis; RA: rheumatoid arthritis; AAU^+^AS^+^: acute anterior uveitis with ankylosing spondylitis; AAU^+^AS^+^: acute anterior uveitis without ankylosing spondylitis; LDR: ligase detection reaction; RFLP: restriction fragment length polymorphism; MALDI-TOF: matrix-assisted laser desorption ionization-time of flight; MS-PCR: methylation-specific polymerase chain reaction; T-ARMS-PCR: tetra amplification refractory mutation system-polymerase chain reaction; MAF: minimal allele frequency; NA: not available; HWE: Hardy Weinberg equilibrium.

**Table 2:** Meta-analysis of miRNA-SNPs with autoimmune diseases.

Subgroup	No of studies	No of case/control	Allele model			Dominant model			Recessive model			Model
			OR (95% CI)	*P*	*P*_H_	OR (95% CI)	*P*	*P*_H_	OR (95% CI)	*P*	*P*_H_	
*miR-146a rs2910164 (associated allele vs. reference allele: G vs. C)*												
All diseases												
Overall	70	20034/28161	1.08 (1.01-1.15)	0.019	<0.001	1.09 (1.01-1.20)	0.049	<0.001	1.09 (0.99-1.19)	0.077	<0.001	R
AITD	3	1103/2322	1.03 (0.93-1.15)	0.57	0.147	1.13 (0.96-1.33)	0.153	0.563	0.94 (0.78-1.13)	0.522	0.171	F
Arthritis	23	5252/6454	1.15 (1.01-1.31)	0.034	<0.001	1.11 (0.95-1.29)	0.158	0.066	1.17 (1.01-1.36)	0.048	0.001	R
Asthma	4	1086/1420	1.16 (1.03-1.31)	0.014	0.314	1.25 (1.02-1.54)	0.029	0.165	1.20 (0.99-1.45)	0.067	0.784	F
SLE	6	2226/3011	1.12 (0.91-1.38)	0.275	0.001	1.15 (0.75-1.76)	0.45	0.002	1.09 (0.86-1.37)	0.49	0.033	R
Uveitis	8	2525/4595	1.44 (1.14-1.81)	0.002	<0.001	1.26 (1.13-1.40)	<0.001	<0.001	1.73 (1.21-2.47)	0.003	<0.001	R
IBD	8	1559/2522	0.79 (0.65-0.97)	0.027	<0.001	0.78 (0.65-0.92)	0.001	0.37	0.67 (0.50-0.88)	0.005	0.015	R
IgA^∗^	2	549/890	0.93 (0.62-1.41)	0.733	0.024	0.79 (0.50-1.24)	0.304	0.082	1.16 (0.47-2.87)	0.752	0.015	R
KD	2	652/742	0.96 (0.82-1.12)	0.596	0.536	0.96 (0.77-1.19)	0.707	0.457	0.93 (0.69-1.25)	0.606	0.882	F
Sclerosis	6	1265/1569	1.04 (0.76-1.42)	0.825	<0.001	1.14 (0.68-1.92)	0.613	0.006	0.96 (0.68-1.36)	0.803	0.011	R
Polymyositis	1	44/107	1.29 (0.76-2.21)	0.350	—	1.76 (0.85-3.65)	0.129	—	0.34 (0.02-6.63)	0.473	—	—
Psoriasis	4	2701/3224	1.15 (1.05-1.25)	0.001	0.269	1.38 (1.13-1.69)	0.002	0.802	1.10 (0.97-1.24)	0.138	0.147	F
T1DM	3	1045/1443	1.09 (0.72-1.64)	0.687	<0.001	1.07 (0.58-1.94)	0.838	0.027	1.12 (0.69-1.82)	0.638	<0.001	R
Ethnicity												
East Asian	29	9164/13636	1.04 (0.96-1.13)	0.353	<0.001	1.04 (0.93-1.16)	0.511	<0.001	1.06 (0.93-1.20)	0.364	<0.001	R
Hispanic	8	2503/3671	1.03 (0.89-1.20)	0.677	0.001	1.05 (0.78-1.41)	0.744	0.012	1.05 (0.88-1.25)	0.576	0.008	R
Middle East	13	1301/1902	1.66 (1.35-2.04)	<0.001	<0.001	2.54 (1.60-4.02)	<0.001	0.015	2.13 (1.54-2.95)	<0.001	<0.001	R
Caucasian	19	6647/8129	0.95 (0.85-1.07)	0.383	<0.001	0.96 (0.83-1.10)	0.539	0.183	0.86 (0.75-0.99)	0.036	0.002	R
Oceanian	1	419/823	0.83 (0.68-1.00)	0.052	—	0.85 (0.52-1.41)	0.535	—	0.78 (0.61-0.99)	0.037	—	—
*miR-196a2 rs11614913 (associated allele vs. reference allele: T vs. C)*												
All diseases												
Overall	26	6555/10237	0.92 (0.88-0.97)	0.001	0.004	0.92 (0.86-0.98)	0.017	0.002	0.87 (0.81-0.95)	0.002	0.046	F
AITD	1	80/486	1.08 (0.77-1.51)	0.677	—	1.11 (0.68-1.82)	0.675	—	1.08 (0.57-2.06)	0.814	—	—
Arthritis	2	507/686	0.99 (0.83-1.18)	0.905	0.3	1.09 (0.86-1.39)	0.454	0.574	0.6 (0.21-1.72)	0.344	0.09	F
Asthma	4	757/980	0.92 (0.80-1.06)	0.249	0.763	0.92 (0.73-1.16)	0.482	0.965	0.84 (0.65-1.09)	0.198	0.518	F
SLE	1	405/486	0.98 (0.81-1.19)	0.831	—	0.97 (0.74-1.27)	0.801	—	0.98 (0.68-1.43)	0.946	—	—
Uveitis	4	1583/2705	0.80 (0.73-0.87)	<0.001	0.661	0.74 (0.64-0.84)	<0.001	0.688	0.74 (0.62-0.87)	<0.001	0.852	F
IBD	9	1948/2906	0.99 (0.88-1.13)	0.97	0.031	1.01 (0.79-1.27)	0.984	0.001	0.98 (0.84-1.12)	0.656	0.18	R
IgA^∗^	1	404/711	0.88 (0.74-1.05)	0.151	—	0.78 (0.57-1.06)	0.106	—	0.9 (0.69-1.17)	0.428	—	—
KD	1	531/623	0.94 (0.80-1.11)	0.489	—	0.99 (0.77-1.28)	0.928	—	0.86 (0.64-1.13)	0.277	—	—
Sclerosis	2	190/504	0.93 (0.72-1.21)	0.6	0.18	1.01 (0.71-1.44)	0.95	0.35	0.69 (0.38-1.25)	0.22	0.125	F
T1DM	1	150/150	1.56 (1.12-2.19)	0.009	—	1.39 (0.88-2.19)	0.163	—	2.64 (1.37-5.09)	0.004	—	—
Ethnicity												
East Asian	12	3899/6447	0.89 (0.84-0.94)	<0.001	0.014	0.87 (0.79-0.96)	0.003	<0.001	0.84 (0.76-0.93)	0.001	0.344	R
Hispanic	3	897/1458	1.02 (0.89-1.15)	0.81	0.87	1.06 (0.88-1.26)	0.551	0.688	0.96 (0.75-1.22)	0.733	0.864	F
Middle East	6	746/958	1.03 (0.89-1.19)	0.652	0.102	1.04 (0.84-1.27)	0.744	0.675	1.01 (0.62-1.65)	0.963	0.022	F
Caucasian	5	1013/1374	0.92 (0.77-1.1)	0.365	0.075	0.89 (0.76-1.06)	0.211	0.314	0.87 (0.58-1.31)	0.502	0.062	R
*miR-499 rs3746444 (associated allele vs. reference allele: C vs. T)*												
All diseases												
Overall	35	8052/12084	1.16 (1.03-1.29)	0.011	<0.001	1.16 (1.09-1.24)	<0.001	<0.001	1.47 (1.3-1.66)	<0.001	<0.001	R
AITD	3	1106/2302	1.16 (0.99-1.34)	0.052	0.923	1.24 (1.04-1.47)	0.015	0.997	0.95 (0.61-1.46)	0.802	0.141	F
Arthritis	12	2075/2933	1.29 (1.15-1.44)	<0.001	<0.001	1.24 (1.09-1.42)	0.001	<0.001	1.54 (1.23-1.92)	<0.001	0.318	R
Asthma	5	1479/1830	1.56 (1.36-1.77)	<0.001	<0.001	1.48 (1.26-1.74)	<0.001	<0.001	2.8 (2.03-3.88)	<0.001	<0.001	R
SLE	3	670/932	1.25 (0.96-1.62)	1.101	0.327	1.31 (0.98-1.73)	0.064	0.328	1.44 (1.01-2.05)	0.044	0.492	F
Uveitis	4	1109/1345	0.83 (0.72-0.97)	0.017	0.089	0.85 (0.71-1.02)	0.089	0.228	0.59 (0.39-0.89)	0.012	0.249	R
IBD	7	1503/2318	1.11 (0.99-1.24)	0.073	0.547	1.02 (0.83-1.25)	0.88	0.053	1.18 (0.65-2.15)	0.594	<0.001	F
Sclerosis	1	110/424	1.15 (0.79-1.67)	0.461	—	1.19 (0.77-1.83)	0.435	—	1.16 (0.31-4.29)	0.823	—	—
Ethnicity												
East Asian	18	5252/7856	1.07 (0.99-1.14)	0.079	0.241	1.03 (0.92-1.16)	0.622	0.012	0.98 (0.67-1.43)	0.916	0.001	R
Hispanic	3	900/1458	1.29 (1.02-1.63)	0.034	0.818	1.31 (1.02-1.68)	0.032	0.71	1.31 (1.02-1.68)	0.034	0.629	F
Middle East	11	1332/1984	1.62 (1.45-1.81)	<0.001	<0.001	1.69 (1.44-1.99)	<0.001	<0.001	2.03 (1.66-2.48)	<0.001	<0.001	R
Caucasian	3	568/786	1.03 (0.85-1.25)	0.729	0.788	1.11 (0.88-1.40)	0.376	0.87	0.77 (0.46-1.29)	0.316	0.784	F

**Table 3:** Metaregression for the heterogeneity of miR-146a rs2910164 comparison in our meta-analysis.

Covariate factors	Allele model				Dominant model				Recessive model			
	Exp (*b*)	Std. Err	*P* value	95% CI	Exp (*b*)	Std. Err	*P* value	95% CI	Exp (*b*)	Std. Err	*P* value	95% CI
Disease subtype	1.23	0.09	0.031	1.02-1.48	1.52	0.24	0.015	1.09-1.23	1.53	0.24	0.017	1.02-1.36
Genotypic method	0.93	0.08	0.377	0.78-1.09	1.02	0.02	0.388	0.98-1.05	1.02	0.02	0.321	0.97-1.06
Ethnicity	2.51	0.74	0.005	1.25-3.42	1.09	0.09	0.264	0.93-1.29	1.19	0.100	0.043	1.01-1.41
Mean age	1.22	0.14	0.083	0.97-1.55	1.12	0.17	0.449	0.83-1.51	1.13	0.17	0.446	0.83-1.53
Number of cases	0.96	0.07	0.589	0.81-1.13	0.99	0.03	0.909	0.94-1.06	1.02	0.04	0.655	0.94-1.11
Percentage of female	1.04	0.11	0.736	0.84-1.28	0.95	0.14	0.736	0.72-1.27	1.11	0.17	0.495	0.82-1.51

Exp (*b*): odds ratio; Std. Err: standard error; CI: confidence interval.

**Table 4:** Bias between miRNA polymorphisms with autoimmune diseases in our meta-analysis.

miRNA polymorphisms	Number of publications	Allele model		Dominant model		Recessive model	
		Begg's test	Egger's test	Begg's test	Egger's test	Begg's test	Egger's test
miR-146a rs2910164	70	0.055	0.286	0.124	0.218	0.235	0.056
miR-196a2 rs11614913	26	0.343	0.061	0.427	0.084	0.930	0.948
miR-499 rs3746444	35	0.680	0.432	0.910	0.910	0.234	0.239
miR-146a rs57095329	18	0.544	0.594	0.443	0.608	0.155	0.825
miR-146a rs2431697	8	0.536	0.498	0.734	0.505	0.308	0.057
miR-146a rs6864584	5	0.142	0.123	0.221	0.120	0.462	0.302
miR-149 rs2292832	9	0.404	0.052	0.251	0.213	0.754	0.820
miR-27a rs895819	6	0.188	0.453	0.707	0.794	0.452	0.214

## Data Availability

The data that support the findings of this study are available from the corresponding author upon reasonable request.

## Supplementary Materials

Supplementary Materials Supplement Table 1: PRISMA 2009 Checklist. Supplement Table 2: meta-analysis of other miRNA-SNPs with autoimmune diseases. Supplement Table 3: the most important findings of the meta-analysis.

## References

[1] Marrack P., Kappler J., Kotzin B. L. (2001). Autoimmune disease: why and where it occurs. *Nature Medicine*.

[2] Hemminki K., Huang W., Sundquist J., Sundquist K., Ji J. (2020). Autoimmune diseases and hematological malignancies: exploring the underlying mechanisms from epidemiological evidence. *Seminars in Cancer Biology*.

[3] Desai M. K., Brinton R. D. (2019). Autoimmune disease in women: endocrine transition and risk across the lifespan. *Frontiers in Endocrinology*.

[4] Hu X., Daly M. (2012). What have we learned from six years of GWAS in autoimmune diseases, and what is next?. *Current Opinion in Immunology*.

[5] Burmester G. R., Pope J. E. (2017). Novel treatment strategies in rheumatoid arthritis. *Lancet*.

[6] Fenoglio C., Cantoni C., de Riz M. (2011). Expression and genetic analysis of miRNAs involved in CD4+ cell activation in patients with multiple sclerosis. *Neuroscience Letters*.

[7] Jimenez-Morales S., Gamboa-Becerra R., Baca V. (2012). MiR-146a polymorphism is associated with asthma but not with systemic lupus erythematosus and juvenile rheumatoid arthritis in Mexican patients. *Tissue Antigens*.

[8] Zhang W., Yi X., Guo S. (2014). A single-nucleotide polymorphism of miR-146a and psoriasis: an association and functional study. *Journal of Cellular and Molecular Medicine*.

[9] Niu Z., Wang J., Zou H., Yang C., Huang W., Jin L. (2015). Common MIR146A polymorphisms in Chinese ankylosing spondylitis subjects and controls. *PLoS One*.

[10] Wang J., Li J., Qiu H. (2019). Association between miRNA-196a2 rs11614913 T&gt;C polymorphism and Kawasaki disease susceptibility in southern Chinese children. *Journal of Clinical Laboratory Analysis*.

[11] Papathanasiou I., Mourmoura E., Balis C., Tsezou A. (2020). Impact of miR-SNP rs2910164 on miR-146a expression in osteoarthritic chondrocytes. *Advances in Medical Sciences*.

[12] Senousy M. A., Shaker O. G., Sayed N. H., Fathy N., Kortam M. A. (2020). LncRNA GAS5 and miR-137 polymorphisms and expression are associated with multiple sclerosis risk: mechanistic insights and potential clinical impact. *ACS Chemical Neuroscience*.

[13] Yenmis G., Soydas T., Ekmekci C. G., Guvercin A. C. Y., Kucuk O. S., Sultuybek G. K. (2020). Fas and microRNAs Variations as a Possible Risk for Behçet Disease. *JCR: Journal of Clinical Rheumatology*.

[14] Xu H. Y., Wang Z. Y., Chen J. F. (2015). Association between ankylosing spondylitis and the miR-146a and miR-499 polymorphisms. *PLoS One*.

[15] Hashemi M., Eskandari-Nasab E., Zakeri Z. (2013). Association of pre-miRNA-146a rs2910164 and premiRNA-499 rs3746444 polymorphisms and susceptibility to rheumatoid arthritis. *Molecular Medicine Reports*.

[16] Xiao C., Rajewsky K. (2009). MicroRNA control in the immune system: basic principles. *Cell*.

[17] Ha T. Y. (2011). The role of microRNAs in regulatory T cells and in the immune response. *Immune Netw*.

[18] Evangelatos G., Fragoulis G. E., Koulouri V., Lambrou G. I. (2019). MicroRNAs in rheumatoid arthritis: from pathogenesis to clinical impact. *Autoimmunity Reviews*.

[19] Mohammadi H., Hemmatzadeh M., Babaie F. (2018). MicroRNA implications in the etiopathogenesis of ankylosing spondylitis. *Journal of Cellular Physiology*.

[20] Pillai R. S., Bhattacharyya S. N., Artus C. G. (2005). Inhibition of translational initiation by Let-7 microRNA in human cells. *Science*.

[21] Humphreys D. T., Westman B. J., Martin D. I., Preiss T. (2005). MicroRNAs control translation initiation by inhibiting eukaryotic initiation factor 4E/cap and poly (A) tail function. *Proceedings of the National Academy of Sciences of the United States of America*.

[22] Nottrott S., Simard M. J., Richter J. D. (2006). Human let-7a miRNA blocks protein production on actively translating polyribosomes. *Nature Structural & Molecular Biology*.

[23] Petersen C. P., Bordeleau M. E., Pelletier J., Sharp P. A. (2006). Short RNAs repress translation after initiation in mammalian cells. *Molecular Cell*.

[24] Chen H. F., Hu T. T., Zheng X. Y. (2013). Association between miR-146a rs2910164 polymorphism and autoimmune diseases susceptibility: a meta-analysis. *Gene*.

[25] Wang D., Pan G. (2019). Association of rs2910164 polymorphism in miRNA-146 and rs3746444 polymorphism in miRNA-499 with inflammatory arthritis: a meta-analysis. *BioMed Research International*.

[26] Li K., Tie H., Hu N. (2014). Association of two polymorphisms rs2910164 in miRNA-146a and rs3746444 in miRNA-499 with rheumatoid arthritis: a meta-analysis. *Human Immunology*.

[27] Dong J., Sun D., Lu F. (2021). Association of two polymorphisms of miRNA-146a rs2910164 (G > C) and miRNA-499 rs3746444 (T > C) with asthma: a meta-analysis. *The Journal of Asthma*.

[28] Landis J. R., Koch G. G. (1977). The measurement of observer agreement for categorical data. *Biometrics*.

[29] Atkins D., Best D., Briss P. A. (2004). Grading quality of evidence and strength of recommendations. *BMJ*.

[30] Higgins J. P., Thompson S. G., Deeks J. J., Altman D. G. (2003). Measuring inconsistency in meta-analyses. *BMJ*.

[31] Tang Z. M., Wang P., Chang P. P. (2015). Association between rs2431697 T allele on 5q33.3 and systemic lupus erythematosus: case-control study and meta-analysis. *Clinical Rheumatology*.

[32] Hruska P., Kuruczova D., Vasku V., Bienertova-Vasku J. (2019). MiR-21 binding site SNP within ITGAM associated with psoriasis susceptibility in women. *PLoS One*.

[33] Che D., Li J., Fu L. (2018). The rs1625579 T&gt;G polymorphism in the <em>miRNA-137</em> gene confers a risk of early-onset Kawasaki disease in a southern Chinese population. *Infect Drug Resist*.

[34] Sheng Y. J., Xu J. H., Wu Y. G. (2015). Association analyses confirm five susceptibility loci for systemic lupus erythematosus in the Han Chinese population. *Arthritis Research & Therapy*.

[35] Yang X. K., Li P., Zhang C. (2017). Association between IRAK1 rs3027898 and miRNA-499 rs3746444 polymorphisms and rheumatoid arthritis: a case control study and meta-analysis. *Zeitschrift für Rheumatologie*.

[36] Zhu M., Li D., Jin M., Li M. (2016). Association between microRNA polymorphisms and the risk of inflammatory bowel disease. *Molecular Medicine Reports*.

[37] Shaker O. G., Abdelaleem O. O., Fouad N. A. (2019). Association BetweenmiR-155, its polymorphism and ischemia-modified albumin in patients with rheumatoid arthritis. *Journal of Interferon & Cytokine Research*.

[38] Labib D. A., Shaker O. G., El Refai R. M., Ghoniem S. A., Elmazny A. (2019). Association betweenmiRNA-146aand polymorphisms of its target Gene,IRAK1, regarding susceptibility to and clinical features of systemic lupus erythematous and multiple sclerosis. *Laboratoriums Medizin*.

[39] Su X. W., Yang Y., Lv M. L. (2011). Association between single-nucleotide polymorphisms in pre-miRNAs and the risk of asthma in a Chinese population. *DNA and Cell Biology*.

[40] Li J., Wang J., Su X. (2020). Association between the miRNA-149 rs2292832 T>C polymorphism and Kawasaki disease susceptibility in a southern Chinese population. *Journal of Clinical Laboratory Analysis*.

[41] Takuse Y., Watanabe M., Inoue N. (2017). Association of IL-10-regulating microRNAs in peripheral blood mononuclear cells with the pathogenesis of autoimmune thyroid disease. *Immunological Investigations*.

[42] Singh S., Rai G., Aggarwal A. (2014). Association of microRNA-146a and its target gene IRAK1 polymorphism with enthesitis related arthritis category of juvenile idiopathic arthritis. *Rheumatology International*.

[43] el-Shal A. S., Aly N. M., Galil S. M., Moustafa M. A., Kandel W. A. (2013). Association of _microRNAs_ genes polymorphisms with rheumatoid arthritis in Egyptian female patients. *Joint, Bone, Spine*.

[44] Zha L., Li S., Liu X. (2019). Association of miR-146a gene polymorphism at loci rs2910164 G/C, rs57095329 A/G, and rs6864584 T/C with susceptibility to Kawasaki disease in Chinese children. *Pediatric Cardiology*.

[45] Lin J., Huang Y., Zhang X., Chen J., Sheng H. (2014). Association of miR-146a rs2910164 with childhood IgA nephropathy. *Pediatric Nephrology*.

[46] Abdelaleem O. O., Fouad N. A., Shaker O. G. (2021). Association of miR-146a rs57095329 with Behçet’s disease and its complications. *British Journal of Biomedical Science*.

[47] Ranjha R., Meena N. K., Singh A., Ahuja V., Paul J. (2017). Association of miR-196a-2 and miR-499 variants with ulcerative colitis and their correlation with expression of respective miRNAs. *PLoS One*.

[48] Shaker O. G., El Boghdady N. A., El Sayed A. E. (2018). Association of MiRNA-146a, MiRNA-499, IRAK1 and PADI4 polymorphisms with rheumatoid arthritis in Egyptian population. *Cellular Physiology and Biochemistry*.

[49] Hu Q., Li B., She R. (2019). Association of polymorphisms of miR-146a rs2910164 locus with clinical features of rheumatoid arthritis. *Zhonghua Yi Xue Yi Chuan Xue Za Zhi*.

[50] Zhang J., Yang B., Ying B. (2011). Association of pre-microRNAs genetic variants with susceptibility in systemic lupus erythematosus. *Molecular Biology Reports*.

[51] Oner T., Yenmis G., Tombulturk K. (2015). Association of pre-miRNA-499 rs3746444 and pre-miRNA-146a rs2910164 polymorphisms and susceptibility to Behcet's disease. *Genetic Testing and Molecular Biomarkers*.

[52] Trinh H. K. T., Pham D. L., Kim S. C., Kim R. Y., Park H. S., Kim S. H. (2017). Association of the miR-196a2, miR-146a, and miR-499 polymorphisms with asthma phenotypes in a Korean population. *Molecular Diagnosis & Therapy*.

[53] Okubo M., Tahara T., Shibata T. (2011). Association study of common genetic variants in pre-microRNAs in patients with ulcerative colitis. *Journal of Clinical Immunology*.

[54] Gazouli M., Papaconstantinou I., Stamatis K. (2013). Association study of genetic variants in miRNAs in patients with inflammatory bowel disease: preliminary results. *Digestive Diseases and Sciences*.

[55] Zare-Karizi S., Vajargah Z. K., Mirfakhraie R. (2018). Association study of miR-146a rs2910164 and rs57095329 polymorphisms with risk of Behcet's disease. *The Journal of Urmia University of Medical Sciences*.

[56] Yang B., Zhang J. L., Shi Y. Y. (2011). Association study of single nucleotide polymorphisms in pre-miRNA and rheumatoid arthritis in a Han Chinese population. *Molecular Biology Reports*.

[57] Inoue Y., Watanabe M., Inoue N. (2014). Associations of single nucleotide polymorphisms in precursor-microRNA (miR)-125a and the expression of mature miR-125a with the development and prognosis of autoimmune thyroid diseases. *Clinical and Experimental Immunology*.

[58] Wang L., Zhang H., Hou N. L. (2020). Correlations of miR-146a and IRAK1 gene polymorphisms with ankylosing spondylitis. *European Review for Medical and Pharmacological Sciences*.

[59] Ridolfi E., Fenoglio C., Cantoni C. (2013). Expression and genetic analysis of MicroRNAs involved in multiple sclerosis. *International Journal of Molecular Sciences*.

[60] Yu H., Liu Y., Zhang L. (2014). FoxO1 gene confers genetic predisposition to acute anterior uveitis with ankylosing spondylitis. *Investigative Ophthalmology & Visual Science*.

[61] Alemán-Ávila I., Jiménez-Morales M., Beltrán-Ramírez O. (2017). Functional polymorphisms inpre-miR146aandpre-miR499are associated with systemic lupus erythematosus but not with rheumatoid arthritis or Graves' disease in Mexican patients. *Oncotarget*.

[62] Luo X., Yang W., Ye D. Q. (2011). A functional variant in microRNA-146a promoter modulates its expression and confers disease risk for systemic lupus erythematosus. *PLoS Genetics*.

[63] Hu D., Zhang Z., Ke X., Kang H., Hong S. (2017). A functional variant of miRNA-149 confers risk for allergic rhinitis and comorbid asthma in Chinese children. *International Journal of Immunogenetics*.

[64] Qi J., Hou S., Zhang Q. (2013). A functional variant of pre-miRNA-196a2 confers risk for Behcet's disease but not for Vogt-Koyanagi-Harada syndrome or AAU in ankylosing spondylitis. *Human Genetics*.

[65] Leng R. X., Wang W., Cen H. (2012). Gene-gene and gene-sex epistatic interactions of MiR146a, IRF5, IKZF1, ETS1 and IL21 in systemic lupus erythematosus. *PLoS One*.

[66] Li Y., Du C., Wang W. (2015). Genetic association of MiR-146a with multiple sclerosis susceptibility in the Chinese population. *Cellular Physiology and Biochemistry*.

[67] Löfgren S. E., Frostegård J., Truedsson L. (2012). Genetic association of miRNA-146a with systemic lupus erythematosus in Europeans through decreased expression of the gene. *Genes and Immunity*.

[68] Ghobadi F., Vaisi-Raygani A., Bahrehmand F. (2017). Genetic variants of pre-microRNAs A-499G(rs3746444) and T-196a2C(rs11614913) with ulcerative colitis (UC) and investigated with thiopurine-S-methyltransferase (TPMT) activity. *Clinical Laboratory*.

[69] Golshani Z., Hojati Z., Sharifzadeh A., Shaygannejad V., Jafarinia M. (2019). Genetic variation in intergenic and exonic miRNA sequence and risk of multiple sclerosis in the Isfahan patients. *Iranian Journal of Allergy, Asthma, and Immunology*.

[70] Yang B., Wei W., Shi Y. (2015). Genetic variation in miR-146a is not associated with susceptibility to IgA nephropathy in adults from a Chinese Han population. *PLoS One*.

[71] Vreca M., Andjelkovic M., Tosic N. (2018). Impact of alterations in X-linked _IRAK1_ gene and _miR-146a_ on susceptibility and clinical manifestations in patients with systemic sclerosis. *Immunology Letters*.

[72] Zhang H., Pu J., Wang X. (2013). IRAK1 rs3027898 C/A polymorphism is associated with risk of rheumatoid arthritis. *Rheumatology International*.

[73] Zhou X., Zhu J., Zhang H., Zhou G., Huang Y., Liu R. (2015). Is the microRNA-146a (rs2910164) polymorphism associated with rheumatoid arthritis? Association of microRNA-146a (rs2910164) polymorphism and rheumatoid arthritis could depend on gender. *Joint, Bone, Spine*.

[74] Zhou Q., Kijlstra A., Hou S. (2012). Lack of association of miR-146a and Ets-1 gene polymorphisms with Fuchs uveitis syndrome in Chinese Han patients. *Molecular Vision*.

[75] Hassine H. B., Boumiza A., Sghiri R. (2017). Micro RNA-146a but not IRAK1 is associated with rheumatoid arthritis in the Tunisian population. *Genetic Testing and Molecular Biomarkers*.

[76] Wei L., Zhou Q., Hou S. (2014). MicroRNA-146a and Ets-1 gene polymorphisms are associated with pediatric uveitis. *PLoS One*.

[77] Zhou Q., Hou S., Liang L. (2014). MicroRNA-146a and Ets-1 gene polymorphisms in ocular Behcet's disease and Vogt-Koyanagi-Harada syndrome. *Annals of the Rheumatic Diseases*.

[78] Srivastava A., Nikamo P., Lohcharoenkal W. (2017). MicroRNA-146a suppresses IL-17-mediated skin inflammation and is genetically associated with psoriasis. *The Journal of Allergy and Clinical Immunology*.

[79] Ibrahim A. A., Ramadan A., Wahby A. A., Hassan M., Soliman H. M., Abdel Hamid T. A. (2019). Micro-RNA 196a2 expression and miR-196a2 (rs11614913) polymorphism in T1DM: a pilot study. *Journal of Pediatric Endocrinology & Metabolism*.

[80] Maharaj A. B., Naidoo P., Ghazi T. (2018). MiR-146a G/C rs2910164 variation in South African Indian and Caucasian patients with psoriatic arthritis. *BMC Medical Genetics*.

[81] Sakoguchi A., Jinnin M., Makino T. (2013). The miR-146a rs2910164 C/G polymorphism is associated with telangiectasia in systemic sclerosis. *Clinical and Experimental Dermatology*.

[82] Okada Y., Jinnin M., Makino T. (2014). MIRSNP rs2910164 of miR-146a is associated with the muscle involvement in polymyositis/dermatomyositis. *International Journal of Dermatology*.

[83] Hussein M. H., Toraih E. A., Aly N. M., Riad E., Fawzy M. S. (2016). A passenger strand variant in miR-196a2 contributes to asthma severity in children and adolescents: a preliminary study. *Biochemistry and Cell Biology*.

[84] Chatzikyriakidou A., Voulgari P. V., Georgiou I., Drosos A. A. (2010). A polymorphism in the 3′-UTR of interleukin-1 receptor-associated kinase (IRAK1), a target gene of miR-146a, is associated with rheumatoid arthritis susceptibility. *Joint, Bone, Spine*.

[85] Assmann T. S., Duarte G. C. K., Brondani L. A. (2017). Polymorphisms in genes encoding miR-155 and miR-146a are associated with protection to type 1 diabetes mellitus. *Acta Diabetologica*.

[86] Ciccacci C., Politi C., Biancone L. (2017). Polymorphisms in MIR122, MIR196A2, and MIR124A genes are associated with clinical phenotypes in inflammatory bowel diseases. *Molecular Diagnosis & Therapy*.

[87] Cai T., Li J., An X. (2017). Polymorphisms in MIR499A and MIR125A gene are associated with autoimmune thyroid diseases. *Molecular and Cellular Endocrinology*.

[88] Ciccacci C., Conigliaro P., Perricone C. (2016). Polymorphisms in STAT-4, IL-10, PSORS1C1, PTPN2 and MIR146A genes are associated differently with prognostic factors in Italian patients affected by rheumatoid arthritis. *Clinical and Experimental Immunology*.

[89] Ahmadi K., Soleimani A., Soleimani Motlagh S., Baharvand Ahmadi S., Almasian M., Kiani A. A. (2020). Polymorphisms of pre-miR-499 rs3746444 T/C and pre-miR-146a rs2910164 C/G in the autoimmune diseases of rheumatoid arthritis and systemic lupus erythematosus in the west of Iran. *Iranian Journal of Public Health*.

[90] Ayeldeen G., Nassar Y., Ahmed H., Shaker O., Gheita T. (2018). Possible use of miRNAs-146a and -499 expression and their polymorphisms as diagnostic markers for rheumatoid arthritis. *Molecular and Cellular Biochemistry*.

[91] Toraih E. A., Ismail N. M., Toraih A. A., Hussein M. H., Fawzy M. S. (2016). Precursor miR-499a variant but not miR-196a2 is associated with rheumatoid arthritis susceptibility in an Egyptian population. *Molecular Diagnosis & Therapy*.

[92] Yu H., Liu Y., Bai L., Kijlstra A., Yang P. (2014). Predisposition to Behçet’s disease and VKH syndrome by genetic variants of miR-182. *Journal of Molecular Medicine (Berlin, Germany)*.

[93] Fattah S. A., Ghattas M. H., Saleh S. M., Abo-Elmatty D. M. (2018). Pre-micro RNA-499 gene polymorphism rs3746444 T/C is associated with susceptibility to rheumatoid arthritis in Egyptian population. *Indian Journal of Clinical Biochemistry*.

[94] Ahmed Ali M., Gamil Shaker O., Mohamed Eid H., Elsayed Mahmoud E., Mahmoud Ezzat E., Nady Gaber S. (2020). Relationship between miR-155 and miR-146a polymorphisms and susceptibility to multiple sclerosis in an Egyptian cohort. *Biomed Rep*.

[95] Caputo V., Strafella C., Termine A. (2020). RNAseq-based prioritization revealed COL6A5, COL8A1, COL10A1 and MIR146A as common and differential susceptibility biomarkers for psoriasis and psoriatic arthritis: confirmation from genotyping analysis of 1417 Italian subjects. *International Journal of Molecular Sciences*.

[96] Chatzikyriakidou A., Voulgari P. V., Georgiou I., Drosos A. A. (2010). The role of microRNA-146a (miR-146a) and its target IL-1R-associated kinase (IRAK1) in psoriatic arthritis susceptibility. *Scandinavian Journal of Immunology*.

[97] Bogunia-Kubik K., Wysoczanska B., Piatek D., Iwaszko M., Ciechomska M., Swierkot J. (2016). Significance of polymorphism and expression of miR-146a and NFkB1 genetic variants in patients with rheumatoid arthritis. *Archivum Immunologiae et Therapiae Experimentalis (Warsz)*.

[98] Xiao M., Ma Y., Chen X., Kuang B. (2015). Single nucleotide polymorphism of miR-149 and susceptibility of rheumatoid arthritis. *Zhong Nan Da Xue Xue Bao. Yi Xue Ban*.

[99] Zhou P. P., Li Y., Ma Z. D., Li Z. Y., Chen F. Y., Jiang Y. X. (2016). Single nucleotide polymorphisms in the promoter region of mir-133a-1 and in pre-mir-152 rs1707 may contribute to the risk of asthma in a Chinese Han population. *European Review for Medical and Pharmacological Sciences*.

[100] Kaidonis G., Gillies M. C., Abhary S. (2016). A single-nucleotide polymorphism in the MicroRNA-146a gene is associated with diabetic nephropathy and sight-threatening diabetic retinopathy in Caucasian patients. *Acta Diabetologica*.

[101] Toraih E. A., Hussein M. H., al Ageeli E. (2017). Structure and functional impact of seed region variant in MIR-499 gene family in bronchial asthma. *Respiratory Research*.

[102] Kiselev I. S., Kulakova O. G., Baulina N. M. (2019). Variability of the MIR196A2 gene as a risk factor in primary-progressive multiple sclerosis development. *Molekuliarnaia Biologiia (Mosk)*.

[103] Zhang L., Wang J., Che D. (2018). The association between the miR-146a rs2910164 C>G polymorphism and Kawasaki disease in a southern Chinese population. *Bioscience Reports*.

[104] Ibrahim W., Sakr B. R., Obaya E., Ghonem H. (2019). MicroRNA-146a expression and microRNA-146a rs2910164 polymorphism in Behcet's disease patients. *Clinical Rheumatology*.

[105] Fouda M. E. (2020). Genetic variants of microRNA-146a gene: an indicator of systemic lupus erythematosus susceptibility, lupus nephritis, and disease activity. *Molecular Biology Reports*.

[106] Massignam E. T., Dieter C., Pellenz F. M., Assmann T. S., Crispim D. (2021). Involvement ofmiR‐126rs4636297 andmiR‐146ars2910164 polymorphisms in the susceptibility for diabetic retinopathy: a case-control study in a type 1 diabetes population. *Acta Ophthalmologica*.

[107] Keewan E., Naser S. A. (2020). MiR-146a rs2910164 G > C polymorphism modulates Notch-1/IL-6 signaling during infection: a possible risk factor for Crohn's disease. *Gut Pathogens*.

[108] Li C., Fu W., Zhang Y. (2015). Meta-analysis of microRNA-146a rs2910164 G>C polymorphism association with autoimmune diseases susceptibility, an update based on 24 studies. *PLoS One*.

[109] Duan R., Pak C., Jin P. (2007). Single nucleotide polymorphism associated with mature miR-125a alters the processing of pri-miRNA. *Human Molecular Genetics*.

[110] Labbaye C., Testa U. (2012). The emerging role of MIR-146A in the control of hematopoiesis, immune function and cancer. *Journal of Hematology & Oncology*.

[111] Jazdzewski K., Liyanarachchi S., Swierniak M. (2009). Polymorphic mature microRNAs from passenger strand of pre-miR-146a contribute to thyroid cancer. *Proceedings of the National Academy of Sciences of the United States of America*.

[112] Park R., Lee W. J., Ji J. D. (2016). Association between the three functional miR-146a single-nucleotide polymorphisms, rs2910164, rs57095329, and rs2431697, and autoimmune disease susceptibility: a meta-analysis. *Autoimmunity*.

[113] Fu L., Jin L., Yan L. (2016). Comprehensive review of genetic association studies and meta-analysis on miRNA polymorphisms and rheumatoid arthritis and systemic lupus erythematosus susceptibility. *Human Immunology*.

[114] The International Consortium for Systemic Lupus Erythematosus Genetics (SLEGEN), Harley J. B., Alarcón-Riquelme M. E. (2008). Genome-wide association scan in women with systemic lupus erythematosus identifies susceptibility variants in _ITGAM_ , _PXK_ , _KIAA1542_ and other loci. *Nature Genetics*.

[115] Yang W., Shen N., Ye D. Q. (2010). Genome-wide association study in Asian populations identifies variants in ETS1 and WDFY4 associated with systemic lupus erythematosus. *PLoS Genetics*.

[116] Hoffman A. E., Zheng T., Yi C. (2009). microRNA miR-196a-2 and breast cancer: a genetic and epigenetic association study and functional analysis. *Cancer Research*.

[117] Sibin M. K., Harshitha S. M., Narasingarao K. V., Dhananjaya I. B., Dhaval P. S., Chetan G. K. (2017). Effect of rs11614913 polymorphism on mature miR196a2 expression and its target gene HOXC8 expression in human glioma. *Journal of Molecular Neuroscience*.

[118] Song G. G., Bae S. C., Seo Y. H. (2015). The association between susceptibility to inflammatory arthritis and miR-146a, miR-499 and IRAK1 polymorphisms. *Zeitschrift für Rheumatologie*.

[119] Ying B., Shi Y., Pan X. (2011). Association of polymorphisms in the human IL-10 and IL-18 genes with rheumatoid arthritis. *Molecular Biology Reports*.

[120] Lin R. J., Lin Y. C. (2010). miR-149∗ induces apoptosis by inhibiting Akt1 and E2F1 in human cancer cells. *Molecular Carcinogenesis*.

[121] Redis R. S., Calin S., Yang Y., You M. J., Calin G. A. (2012). Cell-to-cell miRNA transfer: from body homeostasis to therapy. *Pharmacology & Therapeutics*.

[122] Santonocito S., Polizzi A., Palazzo G., Isola G. (2021). The emerging role of microRNA in periodontitis: pathophysiology, clinical potential and future molecular perspectives. *International Journal of Molecular Sciences*.

