# Supplementary material for: Meta-Analysis of miRNA Variants Associated with Susceptibility to Autoimmune Disease
Source: Dis Markers. 2021 Oct 8;2021:9978460. doi: 10.1155/2021/9978460 (PMC8519726; doi:10.1155/2021/9978460)
Supplement: Supplementary Materials — Supplement Table 1: PRISMA 2009 Checklist. Supplement Table 2: meta-analysis of other miRNA-SNPs with autoimmune diseases. Supplement Table 3: the most important findings of the meta-analysis. [file 9978460.f1.zip › Supplement Materials Table 2 (1).docx]

Meta-analysis of other miRNA-SNPs with autoimmune diseases.

| Subgroup | No of studies | No of Case  /Control | Allele model | | | | Dominant model | | | | Recessive model | | | Model |
| --- | --- | --- | --- | --- | --- | --- | --- | --- | --- | --- | --- | --- | --- | --- |
|  |  |  | OR (95% CI) | P | P_H_ | OR (95% CI) | | P | P_H_ | OR (95% CI) | | P | P_H_ |  |
| miR-146a rs57095329 (Associated allele vs. Reference allele: G vs A) | | | | | | | | | | | | | | |
| All diseases | | | | | | | | | | | | | | |
| Overall | 18 | 10690/12837 | 1.09(1.05-1.15) | <0.001 | <0.001 | 0.93(0.79-1.09) | | 0.402 | <0.001 | 0.85(0.69-1.04) | | 0.114 | 0.441 | R |
| AITD | 2 | 1028/1842 | 1.002(0.87-1.15) | 0.973 | 0.561 | 1.04(0.88-1.22) | | 0.657 | 0.534 | 0.82(0.55-1.23) | | 0.333 | 0.884 | F |
| arthritis | 1 | 608/613 | 1.04(0.85-1.27) | 0.723 | -- | 1.07(0.85-1.36) | | 0.559 | -- | 0.89(0.5-1.57) | | 0.683 | -- | -- |
| asthma | 1 | 124/206 | 0.98(0.65-1.47) | 0.923 | -- | 0.96(0.59-1.54) | | 0.865 | -- | 1.11(0.31-4.02) | | 0.872 | -- | -- |
| SLE | 6 | 5999/5631 | 1.26(1.17-1.35) | <0.001 | 0.141 | 1.14(0.81-1.61) | | 0.461 | -- | 6.37(0.31-132.9) | | 0.232 | -- | F |
| uveitis | 5 | 2172/3699 | 1.21(0.85-1.72) | 0.301 | <0.001 | 0.94(0.7-1.27) | | 0.704 | 0.002 | 1.02(0.72-1.45) | | 0.918 | 0.364 | R |
| KD | 1 | 120/126 | 1.17(0.76-1.82) | 0.471 | -- | 1.29(0.77-2.16) | | 0.341 | -- | 0.87(0.26-2.93) | | 0.821 | -- | -- |
| sclerosis | 2 | 639/720 | 0.56(0.25-1.29) | 0.175 | <0.001 | 0.51(0.18-1.44) | | 0.206 | <0.001 | 0.54(0.33-0.88) | | 0.014 | 0.126 | R |
| Ethnicity | | | | | | | | | | | | | | |
| East Asian | 13 | 9173/11004 | 1.09(1.05-1.15) | 0.001 | <0.001 | 0.98(0.87-1.11) | | 0.77 | 0.037 | 0.88(0.71-1.09) | | 0.23 | 0.854 | R |
| Middle East | 4 | 409/423 | 0.36(0.24-0.55) | <0.001 | <0.001 | 0.29(0.18-0.5) | | <0.001 | <0.001 | 0.23(0.07-0.81) | | 0.022 | <0.001 | R |
| Caucasian | 1 | 1108/1410 | 1.17(0.84-1.64) | 0.359 | -- | 1.14(0.81-1.61) | | 0.461 | -- | 6.37(0.31-132.9) | | 0.232 | -- | -- |
| miR-146a rs2431697 (Associated allele vs. Reference allele: C vs T) | | | | | | | | | | | | | | |
| All diseases | | | | | | | | | | | | | | |
| Overall | 8 | 7472/12932 | 0.77(0.71-0.84) | <0.001 | 0.058 | 0.74(0.56-0.98) | | 0.037 | 0.016 | 0.76(0.62-0.92) | | 0.006 | 0.651 | R |
| arthritis | 1 | 609/616 | 0.98(0.79-1.20) | 0.811 | -- | 1.02(0.80-1.29) | | 0.891 | -- | 0.71(0.38-1.33) | | 0.285 | -- | -- |
| SLE | 7 | 6863/12316 | 0.76(0.71-0.80) | <0.001 | 0.219 | 0.70(0.60-0.81) | | <0.001 | 0.171 | 0.76(0.62-0.94) | | 0.011 | 0.451 | F |
| Ethnicity | | | | | | | | | | | | | | |
| East Asian | 6 | 6303/11500 | 0.77(0.69-0.85) | <0.001 | 0.074 | 0.83(0.54-1.27) | | 0.394 | 0.037 | 0.65(0.38-1.11) | | 0.116 | 0.65 | R |
| Caucasian | 1 | 1104/1392 | 0.81(0.72-0.91) | <0.001 | -- | 0.73(0.62-0.86) | | <0.001 | -- | 0.79(0.64-0.99) | | 0.041 | -- | -- |
| Middle East | 1 | 65/40 | 0.48(0.27-0.84) | 0.010 | -- | 0.28(0.10-0.77) | | 0.013 | -- | 0.48(0.19-1.21) | | 0.120 | -- | -- |
| miR-146a rs6864584 (Associated allele vs. Reference allele: C vs T) | | | | | | | | | | | | | | |
| All diseases | | | | | | | | | | | | | | |
| Overall | 5 | 2186/3800 | 0.83(0.69-0.99) | 0.038 | 0.323 | 0.82(0.68-0.99) | | 0.039 | 0.48 | 0.81(0.35-1.86) | | 0.613 | 0.698 | F |
| asthma | 1 | 124/206 | 0.95(0.5-1.79) | 0.864 | -- | 0.95(0.48-1.88) | | 0.889 | -- | 0.83(0.07-9.24) | | 0.879 | -- | -- |
| uveitis | 3 | 1942/3468 | 0.78(0.64-0.94) | 0.011 | 0.764 | 0.78(0.63-0.95) | | 0.014 | 0.797 | 0.59(0.22-1.65) | | 0.323 | 0.789 | F |
| KD | 1 | 120/126 | 1.59(0.81-3.15) | 0.18 | -- | 1.48(0.72-3.05) | | 0.287 | -- | 5.34(0.25-112.3) | | 0.281 | -- | -- |
| miR-149 rs2292832 (Associated allele vs. Reference allele: C vs T) | | | | | | | | | | | | | | |
| All diseases | | | | | | | | | | | | | | |
| Overall | 9 | 2454/3497 | 1.15(1.06-1.24) | 0.001 | <0.001 | 1.13(1.01-1.26) | | 0.027 | <0.001 | 1.29(0.94-1.79) | | 0.108 | <0.001 | R |
| arthritis | 1 | 186/120 | 2.61(1.85-3.69) | <0.001 | -- | 5.19(3.15-8.56) | | <0.001 | -- | 1.51(0.85-2.66) | | 0.158 | -- | -- |
| asthma | 2 | 284/665 | 2.0(1.01-3.99) | 0.049 | 0.002 | 2.05(0.84-5.01) | | 0.114 | 0.005 | 2.69(1.13-6.44) | | 0.026 | 0.02 | R |
| uveitis | 3 | 1009/1200 | 1.03(0.91-1.18) | 0.623 | 0.668 | 0.99(0.84-1.17) | | 0.896 | 0.65 | 1.18(0.91-1.53) | | 0.224 | 0.847 | F |
| IBD | 2 | 468/900 | 0.79(0.66-0.94) | 0.009 | 0.191 | 0.76(0.61-0.95) | | 0.017 | 0.253 | 0.73(0.49-1.07) | | 0.11 | 0.384 | F |
| KD | 1 | 507/612 | 1.14(0.92-1.4) | 0.229 | -- | 1.14(0.89-1.47) | | 0.308 | -- | 1.17(0.77-1.79) | | 0.461 | -- | -- |
| miR-27a rs895819 (Associated allele vs. Reference allele: G vs A) | | | | | | | | | | | | | | |
| All diseases | | | | | | | | | | | | | | |
| Overall | 6 | 1528/2652 | 1.11(1.01-1.22) | 0.043 | 0.741 | 1.07(0.94-1.22) | | 0.296 | 0.75 | 1.28(1.05-1.55) | | 0.013 | 0.776 | F |
| AITD | 2 | 258/152 | 1.18(0.88-1.58) | 0.272 | 0.832 | 1.09(0.72-1.65) | | 0.679 | 0.526 | 1.64(0.89-3.04) | | 0.115 | 0.706 | F |
| uveitis | 4 | 1270/2500 | 1.09(0.99-1.22) | 0.079 | 0.478 | 1.07(0.93-1.23) | | 0.335 | 0.519 | 1.24(1.01-1.52) | | 0.038 | 0.642 | F |
| miR-155 rs767649 (Associated allele vs. Reference allele: A vs T) | | | | | | | | | | | | | | |
| All diseases | | | | | | | | | | | | | | |
| Overall | 4 | 763/701 | 1.28(0.53-3.06) | 0.587 | <0.001 | 1.45(0.45-4.69) | | 0.539 | <0.001 | 5.48(2.86-10.4) | | <0.001 | 0.269 | R |
| arthritis | 1 | 79/78 | 3.19(2.01-5.06) | <0.001 | -- | 5.13(2.24-11.7) | | <0.001 | -- | 9.08(2.99-27.5) | | <0.001 | -- | -- |
| sclerosis | 2 | 194/232 | 1.14(0.26-5.04) | 0.864 | 0.009 | 1.26(0.2-7.84) | | 0.808 | 0.005 | 4.42(2.32-8.44) | | <0.001 | -- | R |
| T1DM | 1 | 490/391 | 0.59(0.39-0.89) | 0.013 | -- | 0.58(0.38-0.88) | | 0.01 | -- | -- | | -- | -- | -- |
| Ethnicity | | | | | | | | | | | | | | |
| Hispanic | 1 | 490/391 | 0.59(0.39-0.89) | 0.013 | -- | 0.58(0.38-0.88) | |  | -- | -- | | -- | -- | -- |
| Middle East | 3 | 273/310 | 1.84(0.91-3.7) | 0.088 | 0.008 | 2.07(0.61-6.96) | | 0.242 | 0.003 | 5.48(2.86-10.4) | | <0.001 | 0.269 | R |
| miR-125a rs12976445 (Associated allele vs. Reference allele: T vs C) | | | | | | | | | | | | | | |
| AITD | 4 | 1338/2078 | 0.8(0.61-1.05) | 0.109 | 0.078 | 0.76(0.56-1.03) | | 0.08 | 0.068 | 1.02(0.52-2.03) | | 0.945 | 0.536 | R |
| miR-182 rs76481776 (Associated allele vs. Reference allele: C vs T) | | | | | | | | | | | | | | |
| uveitis | 4 | 2190/4900 | 1.62(1.43-1.82) | <0.001 | 0.489 | 1.63(1.43-1.86) | | <0.001 | 0.247 | 2.22(1.48-3.34) | | <0.001 | 0.931 | F |
| miR-585 rs62376935 (Associated allele vs. Reference allele: T vs C) | | | | | | | | | | | | | | |
| uveitis | 3 | 1009/1200 | 1.12(0.98-1.27) | 0.093 | 0.476 | 1.17(0.99-1.39) | | 0.07 | 0.522 | 1.09(0.84-1.42) | | 0.527 | 0.655 | F |
| miR-23a rs3745453 (Associated allele vs. Reference allele: T vs C) | | | | | | | | | | | | | | |
| sclerosis | 2 | 479/500 | 1.68(1.39-2.05) | <0.001 | 0.843 | 1.61(1.25-2.08) | | <0.001 | 0.43 | 2.82(1.84-4.32) | | <0.001 | 0.611 | F |
| miR-106a rs3747440 (Associated allele vs. Reference allele: G vs C) | | | | | | | | | | | | | | |
| AITD | 2 | 317/168 | 1.48(0.85-2.59) | 0.167 | 0.935 | 1.57(0.86-2.88) | | 0.142 | 0.964 | 1.07(0.19-5.91) | | 0.937 | 0.886 | F |
| miR-122 rs17669 (Associated allele vs. Reference allele: C vs T) | | | | | | | | | | | | | | |
| IBD | 2 | 456/452 | 0.87(0.7-1.07) | 0.178 | 0.721 | 0.79(0.61-1.03) | | 0.085 | 0.952 | 1.01(0.62-1.66) | | 0.958 | 0.48 | F |
| miR-124a rs531564 (Associated allele vs. Reference allele: C vs G) | | | | | | | | | | | | | | |
| IBD | 2 | 429/394 | 1.34(0.96-1.88) | 0.087 | 0.831 | 1.29(0.9-1.85) | | 0.161 | 0.737 | 5.6(0.67-46.7) | | 0.111 | 0.842 | F |
| miR-137 rs1625579 (Associated allele vs. Reference allele: G vs T) | | | | | | | | | | | | | | |
| All diseases | | | | | | | | | | | | | | |
| Overall | 2 | 635/726 | 0.88(0.43-1.82) | 0.735 | 0.006 | 0.82(0.36-1.9) | | 0.65 | 0.009 | 1.1(0.21-5.84) | | 0.91 | 0.077 | R |
| KD | 1 | 527/622 | 1.26(0.91-1.74) | 0.159 | -- | 1.23(0.87-1.73) | | 0.238 | -- | 2.97(0.57-15.4) | | 0.194 | -- | -- |
| sclerosis | 1 | 108/104 | 0.6(0.39-0.92) | 0.018 | -- | 0.52(0.3-0.9) | | 0.03 | -- | 0.53(0.2-1.41) | | 0.203 | -- | -- |

**Subgroup meta-analysis by methodologic quality of the studies for ADs as ranked by the NOS scale (rs2910164)**


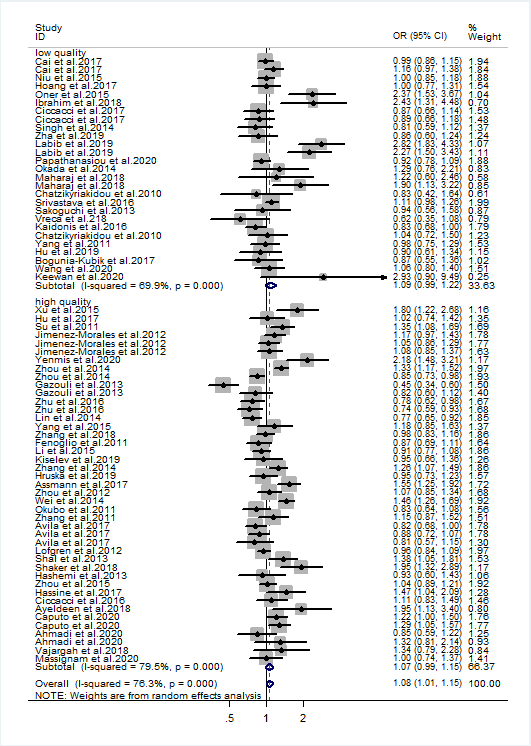


**Subgroup meta-analysis by methodologic quality of the studies for ADs as ranked by the NOS scale (rs11614913)**


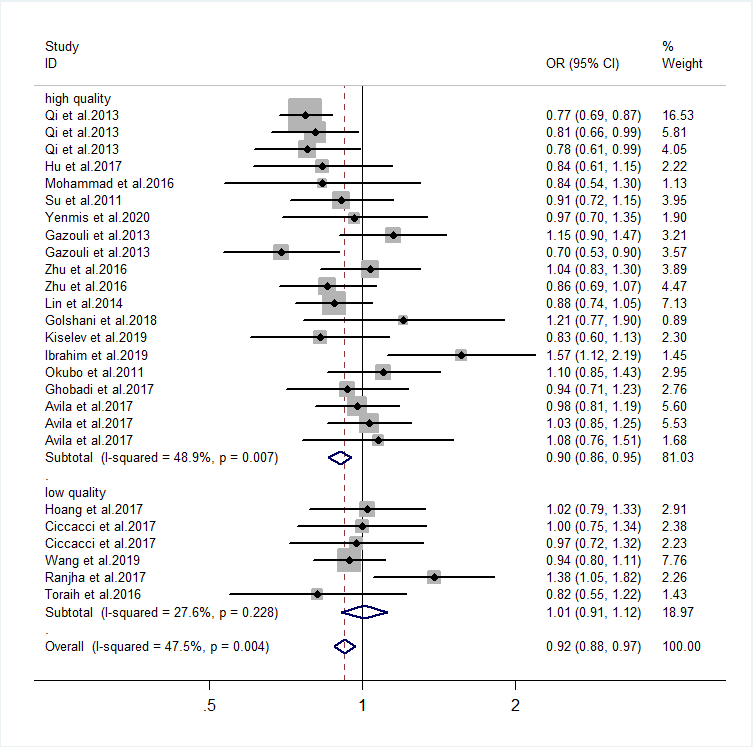


**Subgroup meta-analysis by methodologic quality of the studies for ADs as ranked by the NOS scale (rs3746444)**

**
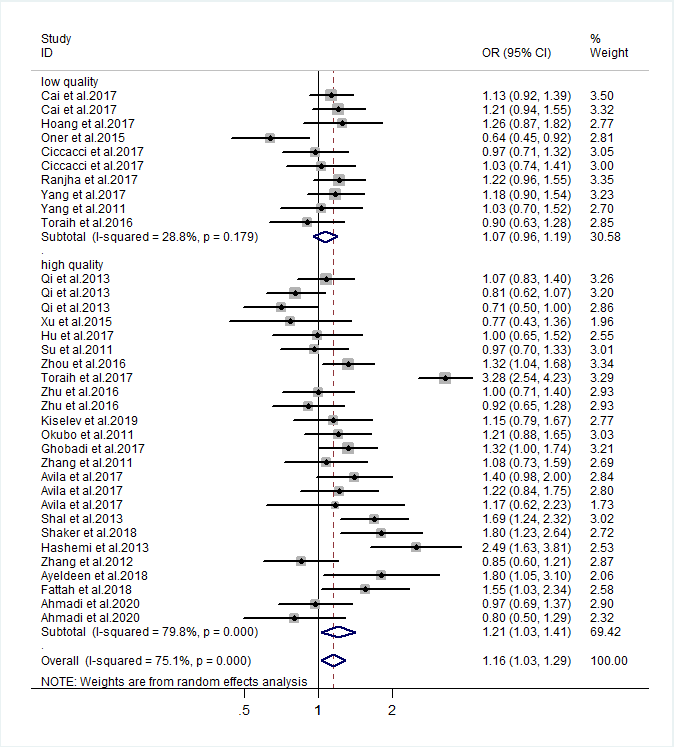
**
