# Supplementary material for: Meta-Analysis of miRNA Variants Associated with Susceptibility to Autoimmune Disease
Source: Dis Markers. 2021 Oct 8;2021:9978460. doi: 10.1155/2021/9978460 (PMC8519726; doi:10.1155/2021/9978460)
Supplement: Supplementary Materials — Supplement Table 1: PRISMA 2009 Checklist. Supplement Table 2: meta-analysis of other miRNA-SNPs with autoimmune diseases. Supplement Table 3: the most important findings of the meta-analysis. [file 9978460.f1.zip › Supplement Materials Table 3.docx]

The most important findings of the meta-analysis.

| miR-146a (rs2910164, rs57095329) was positively associated with AD susceptibility in the overall population. |
| --- |
| miR-146a (rs2431697, rs6864584) was associated with a significantly decreased risk of AD in the overall population. |
| miR-196a2 rs11614913 was associated with a lower risk of AD in the overall population. |
| miR-499 rs3746444 is a predisposing cause of AD in the overall population. |
| miR-149 rs2292832 conferred a significantly increased risk of AD in the overall population. |
| miR-27a rs895819 conveyed a significantly elevated susceptibility to AD in the overall population. |
